# Natural and Pathological Aging Distinctively Impacts the Pheromone Detection System and Social Behavior

**DOI:** 10.1007/s12035-023-03362-3

**Published:** 2023-05-02

**Authors:** Adrián Portalés, Pablo Chamero, Sandra Jurado

**Affiliations:** 1 Instituto de Neurociencias de Alicante, Consejo Superior de Investigaciones Científicas - Universidad Miguel Hernández (CSIC-UMH), 03550 Sant Joan d´Alacant, Spain; 2 Laboratoire de Physiologie de La Reproduction Et Des Comportements, CNRS, IFCE, INRAE, University of Tours, 37380 Nouzilly, France

**Keywords:** Aging, Pheromone, Vomeronasal system, Social behavior, Odor discrimination, Neurodegeneration

## Abstract

Normal aging and many age-related disorders such as Alzheimer’s disease cause deficits in olfaction; however, it is currently unknown how natural and pathological aging impacts the detection of social odors which might contribute to the impoverishment of social behavior at old age further worsening overall health. Analysis of the vomeronasal organ, the main gateway to pheromone-encoded information, indicated that natural and pathological aging distinctively affects the neurogenic ability of the vomeronasal sensory epithelium. Whereas cell proliferation remained majorly preserved in 1-year-old APP/PS1 mice, naturally aged animals exhibited significant deficiencies in the number of mature, proliferative, and progenitor cells. These alterations may support age-related deficits in the recognition of social cues and the display of social behavior. Our findings indicate that aging disrupts the processing of social olfactory cues decreasing social odor exploration, discrimination, and habituation in both wild-type senescent (2-year-old) mice and in 1-year-old double mutant model of Alzheimer’s disease (APP/PS1). Furthermore, social novelty was diminished in 1-year-old APP/PS1 mice, indicating that alterations in the processing of social cues are accelerated during pathological aging. This study reveals fundamental differences in the cellular processes by which natural and pathological aging disrupts the exploration of social information and social behavior.

**Supplementary Information:**

The online version contains supplementary material available at 10.1007/s12035-023-03362-3.

## Introduction

Social recognition is essential for survival allowing to appropriately adapt behavior across a variety of contexts [1, 2]. As aging progresses, social odors have been shown to elicit attenuated responses [3], a phenomenon interpreted as a natural consequence of age-related decline in sensory perception. In humans, social impoverishment has been identified as a major aggravating factor for decreased life expectancy [4, 5] and an indicator of the appearance of dementia and neurodegenerative disorders such as Alzheimer’s disease (AD) [6, 7]. Despite the central role of social interaction in maintaining overall well-being, the mechanisms by which aging, natural or pathological, alters social information processing remain unclear.

Mouse social communication strongly depends on chemosignals comprised by volatile and non-volatile molecules (pheromones) [8, 9]. Volatile odors are primarily detected at the main olfactory epithelium (MOE) which projects to the main olfactory bulb (MOB), whereas pheromones are mainly detected by the vomeronasal sensory epithelium (VSE) [10–12] which connects to the accessory olfactory bulb (AOB) [13]. Despite the segregated connectivity, both systems have overlapping functions [14, 15].

A remarkable property of the VSE is that it contains proliferative niches capable of generating functional neurons during adulthood [16–19]. The neurogenic properties of olfactory structures are known to decline with age, which it is likely to underlie the olfactory deficits associated to both natural aging and neurodegenerative disorders [20–22]. However, how natural and pathological aging affects the regenerative capacity of the VSE has been scarcely studied despite being central to socio-sexual cue detection. An elegant study by Brann and Firestein [18] showed that the proliferative capacity of the marginal region of the VSE of 2-year-old animals was attenuated in comparison to young animals. More recently, Mechin et al. (2021) [23] reported a significant reduction in mature olfactory marker protein (OMP)-expressing cells (OMP^+^), indicating structural modifications of the aged VNO. However, it is currently unclear whether these cell alterations have any impact in social odor detection, particularly in animal models of neurodegeneration.

To gain insight into these questions, we investigated the impact of natural and pathological aging in social odor-evoked sniffing behavior, social-odor habituation/dishabituation, and sociability during the aging process of wild-type C57/BL6 mice and amyloid precursor protein (APP) and presenilin 1 (PS1) double transgenic mice (APP/PS1^Het^ mice, therein), a widely used animal model of AD [24–26]. Analysis of the number of progenitors, proliferative and mature neurons revealed that naturally aged animals show reduced neuronal proliferation and decreased levels of sensory mature OMP^+^ neurons. In contrast, the VSE of 1-year-old APP/PS1^Het^ mice exhibited normal levels of mature neurons and cell regeneration, suggesting that natural and pathological aging distinctively impacts the neurogenic capacity of the VSE.

We explored the functional consequences of these cell alterations observing reduced exploration time of social odors and decreased performance in a social odor habituation-dishabituation test in both senescent and middle-aged APP/PS1^Het^ mice, suggesting that despite the overall normal VSE structure and cell composition, APP/PS1^Het^ animals exhibit an accelerated decay of social cue exploration. Moreover, 1-year-old APP/PS1^Het^ mice exhibited a significant reduction of social novelty which was not apparent in naturally aged animals, exposing fundamental differences in how natural and pathological aging impacts not only the pheromone detection system but also the display of social behavior.

## Materials and Methods

### Animals

Experiments were performed in either control C57/BL6 mice (young adult: 2–4 months old; middle age: 6–8 months old; old: 12–14 months old; senescent: 20–24 months old) or APP/PS1 mice on a C57/BL6 genetic background (young adult: 2–4 months old; old: 12–14 months old) (Jackson Labs, Stock No. 004462, MMRRC Stock No. 34829). Distinct animals were utilized in each of the test conducted. APP/PS1^Het^ mice express a chimeric mouse/human amyloid precursor protein (Mo/HuAPP695swe) and a mutant human presenilin 1 (PS1-dE9). These animals develop beta-amyloid deposits at ~ 6 months of age and exhibit early-onset cognitive impairments [24–26] and reduced life expectancy (~ 14–16 months old) in comparison to APP/PS1^WT^ controls, a scenario that prevented the study’s extension to a comparable age range to naturally aged animals (20–24 months old). Mice were kept group-housed and experimentally naïve during all their lifetime under pathogen-free conditions. Animals were housed in ventilated cages with free access to food and water on a 12 h light/dark cycle. Although the study was not originally designed to address sexual differences, many experiments included animals of both sexes. However, a meaningful statistical comparison of the results desegregated by sex could be only performed for the social exploration test.

All experiments were performed according to Spanish and European Union regulations regarding animal research (2010/63/EU), and the experimental procedures were approved by the Bioethical Committee at the Instituto de Neurociencias and the Consejo Superior de Investigaciones Científicas (CSIC).

### VNO Dissection

Mice were perfused transcardially with PBS, pH 7.4, followed by 4% paraformaldehyde in 0.1 M phosphate buffer (PB), pH 7.4. A fixed mouse head was placed under a scope, and the lower jaw was removed to get a palate view. To facilitate VNO extraction, the palate and the nasal septum were removed and the bilateral VNOs were split in two parts with a gentle twisting motion. Finally, the bony capsule that covers each portion was carefully removed to extract the VNOs. Tissue was incubated in a 30% sucrose solution for cryoprotection and kept at − 4 °C until sectioning. Samples were embedded in OCT and frozen at − 80 °C for cryosectioning.

### Immunohistochemistry

VNOs embedded in OCT medium were sliced in 16 μm thick sections in a cryostat apparatus (Leica CM 3050S). Slices were incubated in blocking solution containing 0.5% Triton X-100 and 5% normal horse serum in 0.1 M TBS for 2 h at room temperature (RT). Primary antibody incubation was performed overnight (o/n) at 4 °C with anti-OMP (Wako goat polyclonal; 1:2000), anti-PCNA (Sigma, rabbit monoclonal; 1:2000), and anti-Sox2 (R&D Systems, goat polyclonal; 1:300). For PCNA immunostaining, slices were incubated in 10 mM citrate buffer (100 °C; pH, 6.0) for 5 min prior staining. Sections were incubated with Alexa Fluor 488- or 594-conjugated secondary antibodies (Jackson Laboratories, 1:500) for 2 h at RT. Hoechst (Sigma, 1:10,000) was added during 5 min after the secondary antibody incubation for nuclei visualization. Imaging was performed using a vertical confocal microscope Leica SPEII. Final images were assembled in Adobe Illustrator.

### Sox2 Fluorescence Intensity at the VNO Supporting Cell Layer (SCL)

For determining the expression of Sox2 in the VNO SCL where single-cell quantification was limited by the densely packed disposition of the cells, we calculated the corrected total fluorescence (CTF) of the area of interest employing the Freehand ROI tool of Image J. CTF was calculated by subtracting the background fluorescence from a minimum of 3 sections, 16 μm thick, from at least 4 animals per condition.

### Stereology, Cell Quantification, and AOB Volume Estimation

The total number of olfactory mature neurons (OMP^+^ neurons) in the entire VSE was estimated using stereology (optical fractionator method [27]) employing Stereo Investigator (MBF Bioscience). Two different tools are combined in this unbiased quantification method: a 3D optical dissector for cell counting and a fractionator, based on a systematic, uniform, and random sampling over a known area [28, 29]. The number of neurons was estimated as

$$N=\left(1/h\times 1/ssf\times 1/asf\times \sum Q-t\right)$$

in which **∑ Q** is the total cell number in the region of interest acquired using the optical dissector method; **t** stands for the mean mounted section thickness; **h** is the optical dissector height; **asf** is the area sampling fraction and **ssf** is the frequency of sampling (section sampling fraction). For the VSE stereological analysis, sampling was performed with a 20 × objective (Leica, NA 0.6), the counting frame area was 2500 μm^2^, and the sampling grid area was 22,500 μm^2^. *H* for VSE stereology was 8 μm with 1 μm as upper and lower guard zones and *t* was set at 10 μm. Quantifications were performed for marginal regions of the VSE dividing the area in segments of equal length as previously described [18, 30]. For cell quantification in the anterior–posterior regions, the VSE was distributed in 10 sections per animal. Results were divided by the area to obtain the number of cells/mm^2^ in each subdivision. Quantification of the cell number in the marginal area was performed in three slices of each animal delineated with a 50 × 50 dissector employing Stereo Investigator (MBF Bioscience).

VSE volume was calculated by multiplying the sampled area by the slice thickness (16 μm) and the number of series (10 slices per animal). Ten sections per series were analyzed to obtain an estimation of the VSE area and volume in young adults (4 months old), senescent (24 months old), and old (12 months old) APP/PS1^WT^ and old APP/PS1^Het^ mice.

The volume of the AOB was estimated by measuring the AOB area and multiplying the sampled area by the section thickness (50 μm) and the number of analyzed Sects. (5 series per animal).

### Odorants

Social and non-social scents were employed to discern the impact of aging in social odor perception. As non-social scents, we used (i) the banana-like odor isoamyl acetate (IA) (Sigma) shown to be primarily detected at the MOE level (Xu et al., 2005) with neutral valence within a broad dilution range [31–33] and (ii) food pellets to perform a food finding test (FFT, see experimental details below). As a social scent, we used urine from conspecifics which has been shown to elicit robust VNO activity [34]. Urine samples were collected according to standard procedures [35]. For habituation-dishabituation tests, frozen urine samples from young cage mates were used to test fine odor discrimination. For sensitivity tests, urine samples were combined in a stock sample used for each round of odor presentations until experiment completion. For female urine, samples obtained at different points of the estrous cycle were pooled to generate a uniform stock.

### Behavioral Assessment

All the behavioral experiments using social and non-social stimuli were performed in a dedicated room with continuous air reposition under dim indirect light (20 lx). Odor dilutions were prepared in a room outside the animal house. Odor presentation was performed in a homemade methacrylate box with removable walls for cleaning. The chamber dimensions were 15 cm width, 15 cm length, and 30 cm high. A small hole (1 cm diameter) was performed in the middle of one of the sides located 5 cm above from the box ground to fit standard cotton sticks impregnated with 1 μL of the odorant allowing direct contact with the stick. Habituation to the testing conditions was performed before odorant presentations consisting of handling (5 min), free exploratory activity in the box and habituation to the cotton stick movement (5 min). Urine was presented embedded in a cotton stick in order to preserve non-volatile pheromones and mimic the natural detection method of physical contact between conspecifics. To avoid variability due to the intensity of the volatile components of the urine, only direct nose contact with the tip of the cotton stick was considered as explorative behavior (sniffing/exploration time). Experiments were monitored by a video recording camera fixed 15 cm above the box. Videos were collected and analyzed offline using SMART video-tracking software (PanLab S.L.).

- Odor exploration test: After a period of habituation (10 min), mice were exposed to two control trials (mineral oil for IA assays or water for urine tests) during 1 min separated by intervals of 1 min to avoid odor habituation [36, 37]. Subsequent presentations consisted on serial dilutions of either urine samples (diluted in water: 1:1.000, 1:500, 1:250, 1:100, 1:50, 1:10, and non-diluted (nd)) or IA were tested in ascending order (diluted in mineral oil: 1:5 × 10^5^, 1:100.000, 1:10.000, 1:1.000, 1:100). Animals were considered to detect the olfactory stimulus when spent more time investigating the odors than the vehicles (water or mineral oil).
- Food finding test (FFT): FFT was performed following standard procedures [38]. The mice were food deprived for 24 h before testing. Five food pellets (~ 35 g) were placed in a corner and covered by 5 cm of litter bedding. Animals were considered to detect the food pellet when spent digging, touching, and holding the food pellet for more than 5 s.
- Social odor habituation-dishabituation test: The effect of aging in social odor discrimination and habituation was explored using an adapted habituation-dishabituation test [39]. After a period of cage habituation (10 min), two control trials were performed employing cotton sticks soaked with 1 μL of water (vehicle). Three successive presentations of urine from an animal of the opposite sex (S1a-c) were followed by three presentations of urine from a different subject of the opposite sex (S2a-c) to evaluate fine chemo-olfactory discrimination and habituation. Urine samples came from young animals of the opposite sex to maximize approaching behavior [3]. Each sample presentation lasted 1 min separated by 1 min intervals. Habituation was estimated as a decrease in the exploration time (sniffing) over the cotton stick tip after the first exposure of urine from the same animal (S1a). Dishabituation (discrimination) occurs in response to a new odor presentation (S2a) as a measurable increase in the exploration time. The test allows to explore two consecutive phases of discrimination (H_2_O^B^-S1a and S1c-S2a) and habituation (S1a-S1b and S2a-S2b). Trend lines between H_2_O^B^-S1a, S1c-S2a, S1a-S1b, and S2a-S2b were fitted to obtain the slope values indicating the amplitude of the discrimination and habituation effect for each tested condition. Higher positive values indicated more pronounced social discrimination, and higher negative values corresponded to more robust habituation.
- Long-term social habituation-dishabituation test: The aforementioned social odor habituation task was adapted to assay social cue memory by presenting the urine sample employed in S1a, 24 h after the first test was performed. Intact social odor memory was manifested as a reduction of the sniffing time during the second presentation of S1a, an effect which was clearly apparent in young animals.
- Three-chamber test: Social testing was performed in a cage (60 × 40 × 22 cm) following standard procedures [40]. Dividing walls were made from clear Plexiglas, with openings allowing access into each chamber. The test mouse was first placed in the middle chamber and allowed to explore for 10 min. After the habituation period, an unfamiliar subject of the same sex (mouse 1, M1^A^) was placed in one of the side chambers. The unfamiliar mouse was enclosed in a small, round wire cage, which allowed nose contact between the bars. In this first session (sociability), the test mouse had a choice of spending time in either the empty chamber (E) or the chamber occupied by M1^A^. At the end of the sociability session, each mouse was tested in a second 10-min session to evaluate social preference for a new subject. A second, unfamiliar mouse (mouse 2, M2) of the same sex was placed in the chamber that had been empty during the first session. This second unfamiliar mouse was enclosed in an identical wire cage than M1^A^. The test mouse had a choice between the first, already-investigated mouse (M1^B^) and the novel unfamiliar mouse (M2) which indicates their social preference or social novelty [40]. Continuous video recordings were collected and analyzed offline using BORIS and SMART video-tracking software (PanLab S.L.). Measures of time spent sniffing E, M1^A−B^, and M2 were quantified for each session.

#### Data Analysis

All data were tested for statistical significance using GraphPad Prism 8. The Shapiro–Wilk test was used to determine data normality. One-way ANOVA with Tukey’s test for multiple comparisons with a single variable was implemented for cell quantifications and anatomical data. For the behavioral analysis, a two-way ANOVA with Tukey’s test was used to test multiple comparisons with more than one variable. *P* values are indicated in all figures above the corresponding comparisons. $P \le 0.05$ was considered statistically significant. *P* values are indicated in all figures above the corresponding comparisons. In addition, a two-way ANOVA with the interaction of age vs. genotype has been applied to all data sets including these variables. Results of these statistical analyses can be found in Supplementary Results. A summary of all data presented in the study with their correspondent *P* values can be found in Supplementary Results. In addition, Supplementary Figures 3, 4, 6, and 7 summarize the *P* and *N* values corresponding to all the statistical comparisons between different ages, genotypes, and dilutions of the social odor sensitivity tests.

## Results

### Structural Modifications of the Mouse VSE During Natural and Pathological Aging

**Fig. 1:**
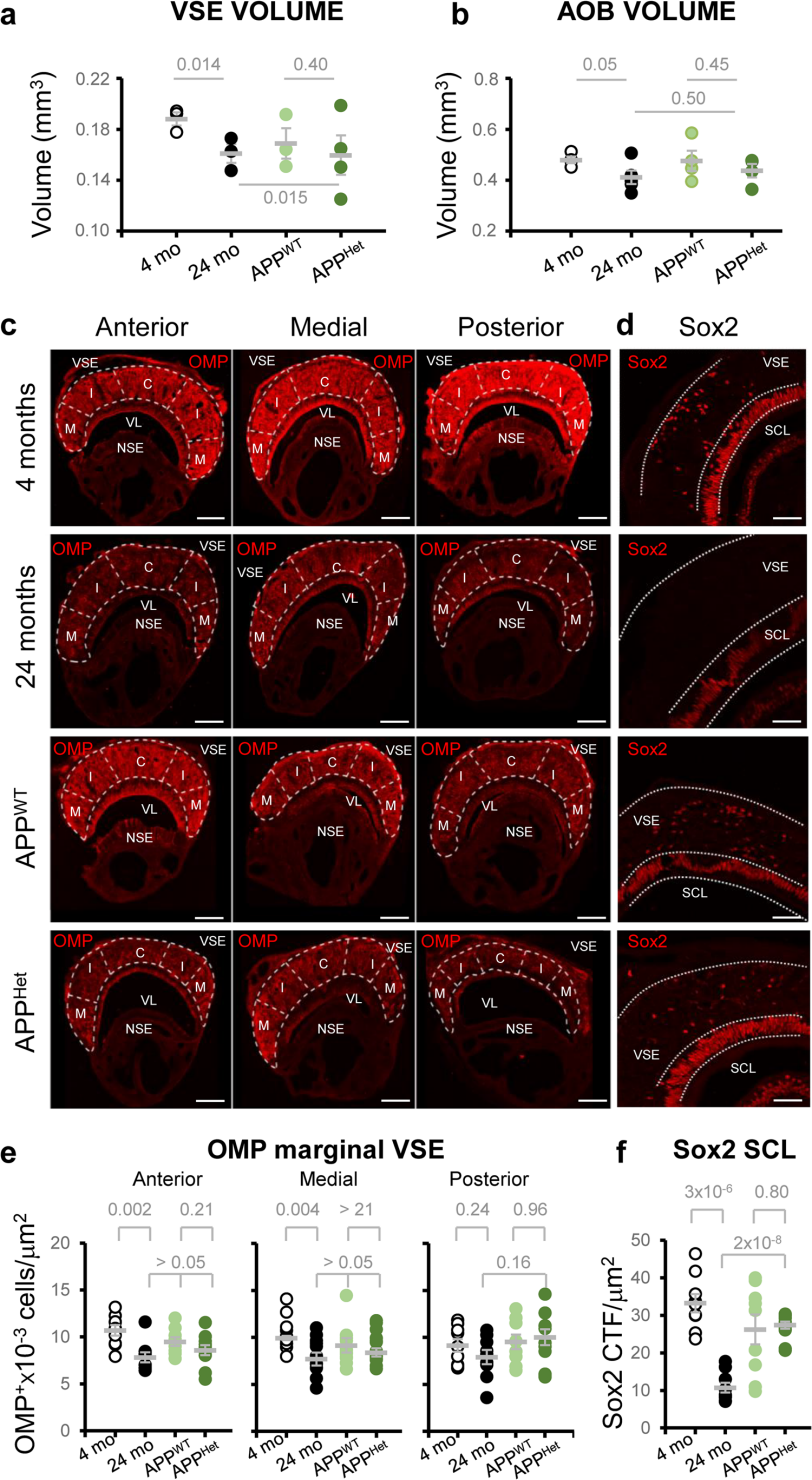
Structural modifications of the mouse VSE during natural and pathological aging. **a** Dispersion plot of the VSE volume indicates a significant reduction during natural but not pathological aging. **b** Dispersion plot of AOB volume. Thick lines indicate the mean ± SEM. Data were obtained from 4 animals per condition. **c** Representative images of OMP staining in the VSE during natural and pathological aging. Scale bar indicates 100 μm. **d** Representative images of Sox2 staining in the VSE and the supporting cell layer. Scale bar indicates 100 μm. VL: vomeronasal lumen; SCL: supporting cell layer; NSE: non-sensory epithelium; M: marginal zone; I: intermediate zone; C: central zone. **e**, **f** Dispersion plots represent the number of OMP^+^ cells in the marginal VSE (number of cells × 10^3^/μm^3^) and the intensity of Sox2 fluorescence in the SCL (Sox2 CTF/μm^2^). Thick lines indicate the mean ± SEM. Data were obtained from slices from at least three animals per condition. Statistical comparisons were calculated by two-way ANOVA with Tukey’s test. $P \le 0.05$ was considered statistically significant. *P* values are indicated above the corresponding comparisons

We sought to investigate the structure of the VSE during natural and pathological aging, as the main gateway of pheromone (non-volatile)-encoded social information [41, 42]. Stereological analysis revealed significantly smaller VSE volumes in 2-year-old (senescent) mice (Fig. 1a; Supplementary Results—Table 1). This observation was supported by the reduced number of olfactory marker protein (OMP)-positive cells (OMP^+^ cells) along the rostrocaudal axis of the marginal VSE (Fig. 1c, e; Supplementary Results—Table 2; Supplementary Figure 1).

We hypothesized that the drop in the number of OMP^+^ cells in senescent mice could translate into a reduced axonal projection to the AOB, the main VNO target area, resulting in smaller AOB volumes (Supplementary Figure 2). As expected, we observed a reduction in AOB size with no apparent histopathological alterations in 2-year-old mice (Fig. 1b; Supplementary Results—Table 3), indicating that natural aging alters basic structural features of the VNO-AOB axis.

We expanded our analysis to the SRY-box transcription factor 2 (Sox2)-expressing cells (Sox2^+^ cells) in the supporting cell layer (SCL), a neuronal stem cell marker which also stains mature differentiated sustentacular cells [43–45]. The estimation of the labeling intensity of Sox2 in the SCL showed a drastic reduction in senescent but not in APP/PS1^Het^ mice (Fig. 1d, f; Supplementary Results—Table 4). These results show that natural aging disrupts VSE structure by reducing the number of sensory (OMP^+^) and SCL sustentacular (Sox2^+^) cells, although this may not imply a total elimination of the proliferative capacities [18].

### Natural and Pathological Aging Differentially Impacts VSE Cell Proliferation

**Fig. 2:**
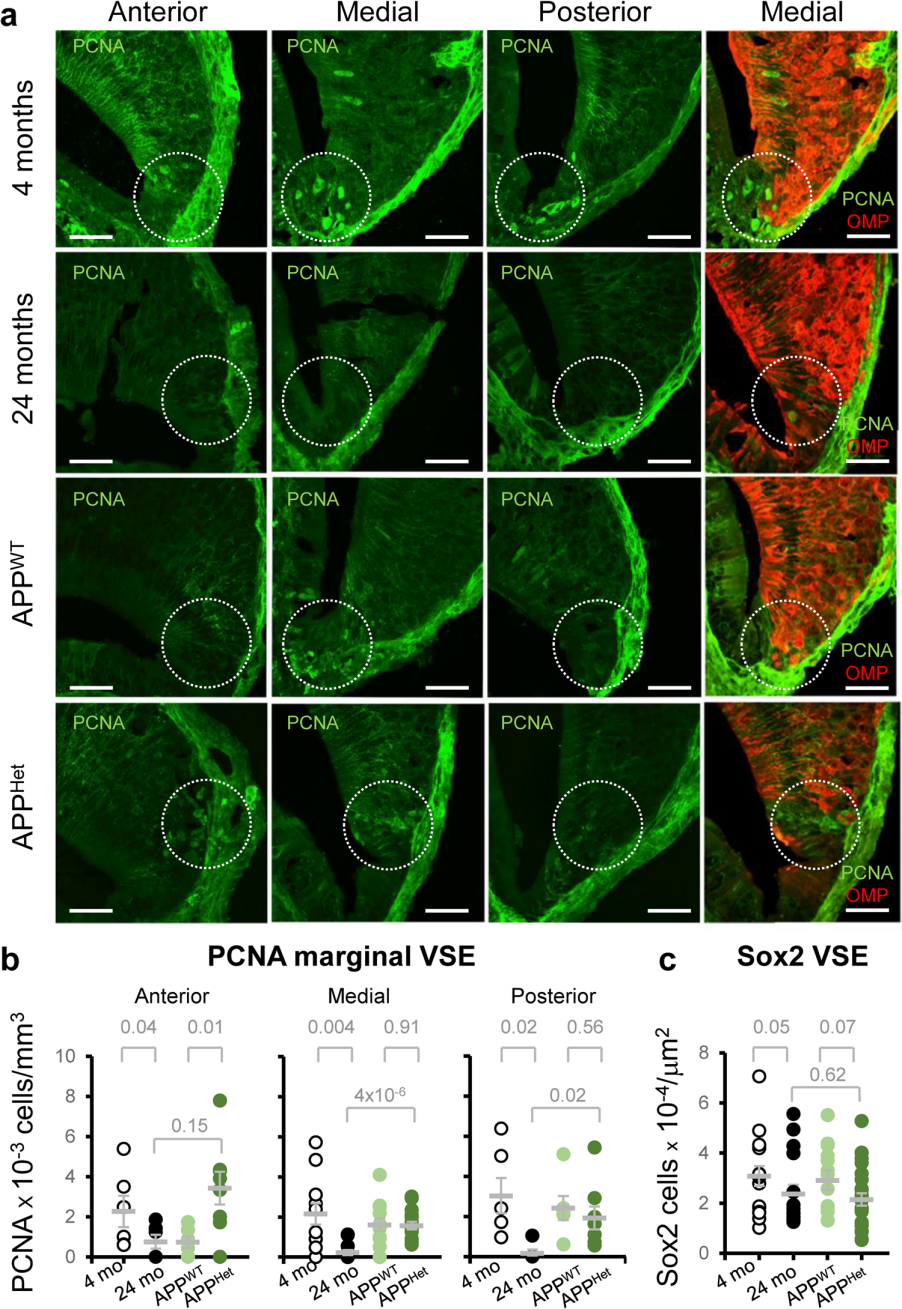
Natural and pathological aging differentially impacts cell proliferation in the marginal VSE. **a** Representative confocal images of PCNA staining in the VSE proliferative niche during natural and pathological aging. Panels on the right show confocal images of OMP and PCNA double staining in the VSE. To note, there is no overlapping signal between the two markers indicating the identification of cells at different maturation stages. Circles indicate active proliferative marginal region of the VSE. Scale bar represents 20 μm. **b**, **c** Dispersion plots represent the number of PCNA^+^ cells (number of cells × 10^−3^/mm^3^) and the number of Sox2^+^ cells (number of cell × 10^−4^/μm^2^) in the marginal VSE. Thick lines indicate the mean ± SEM. Data were obtained from slices from at least three animals per condition. Statistical analysis was calculated by two-way ANOVA with Tukey’s test for multiple comparisons. $P \le 0.05$ was considered statistically significant. *P* values are indicated above the corresponding comparisons

Because of the lack of data on the characteristics of the VSE neurogenic niche in animal models of neurodegeneration, we conducted an analysis to examine the expression of proliferative cell nuclear antigen (PCNA)-positive cells (PCNA^+^ cells) in the anterior, medial, and posterior VNO of senescent and APP/PS1^Het^ mice. Consistent with previous reports, cell proliferation in young animals was abundant in the marginal zone of the anterior and medial VSE [18, 30] (Fig. 2a, b). Senescent mice exhibited a significant reduction in PCNA^+^ cells in the marginal VSE (Fig. 2a, b; Supplementary Results—Table 5), indicating reduced cell proliferation. Surprisingly, the number of PCNA^+^ cells in APP/PS1^Het^ mice was significantly higher in the anterior VSE whereas it showed a downward trend in the posterior VNE (Fig. 2a, b), suggesting a region-specific increase in cell proliferation in these animals. Importantly, no significant overlap between OMP and PCNA staining was observed which demonstrates that these two markers recognize cell populations at different maturation stages (OMP-PCNA double immunostaining, right panel in Fig. 2a).

We then sought to explore whether the reduced number of proliferative PCNA^+^ cells in senescent mice could be due to a decrease of stem cell generation by analyzing the number of Sox2^+^ neural precursor cells in the VSE [46, 47]. We observed a significant reduction in the number of Sox2^+^ cells in 2-year-old mice but not in APP/PS1^Het^ animals, indicating reduced neurogenic capacity in the naturally aged VSE (Fig. 2c; Supplementary Results—Table 6). Altogether, these findings revealed a fundamental difference in how natural and diseased aging impacts the VSE structure and proliferative capacity.

### Late Onset of Social Exploration Deficits During Natural Aging

Our data indicated that natural aging induces significant alterations in the structure, VSE proliferative capacity, and cellular composition; thus, we investigated whether these adaptations might translate into impairments in the processing of socio-sexual information. Cumulative evidence points to olfactory decline as a common symptom of natural and pathological aging [4, 5, 48, 49]. However, most of these studies and diagnostic tests employed synthetic odors with reduced social valence; therefore, the temporal course and severity of the age-related involution of the recognition of social cues, largely processed by the VNO-AOB system, remain unclear.

**Fig. 3:**
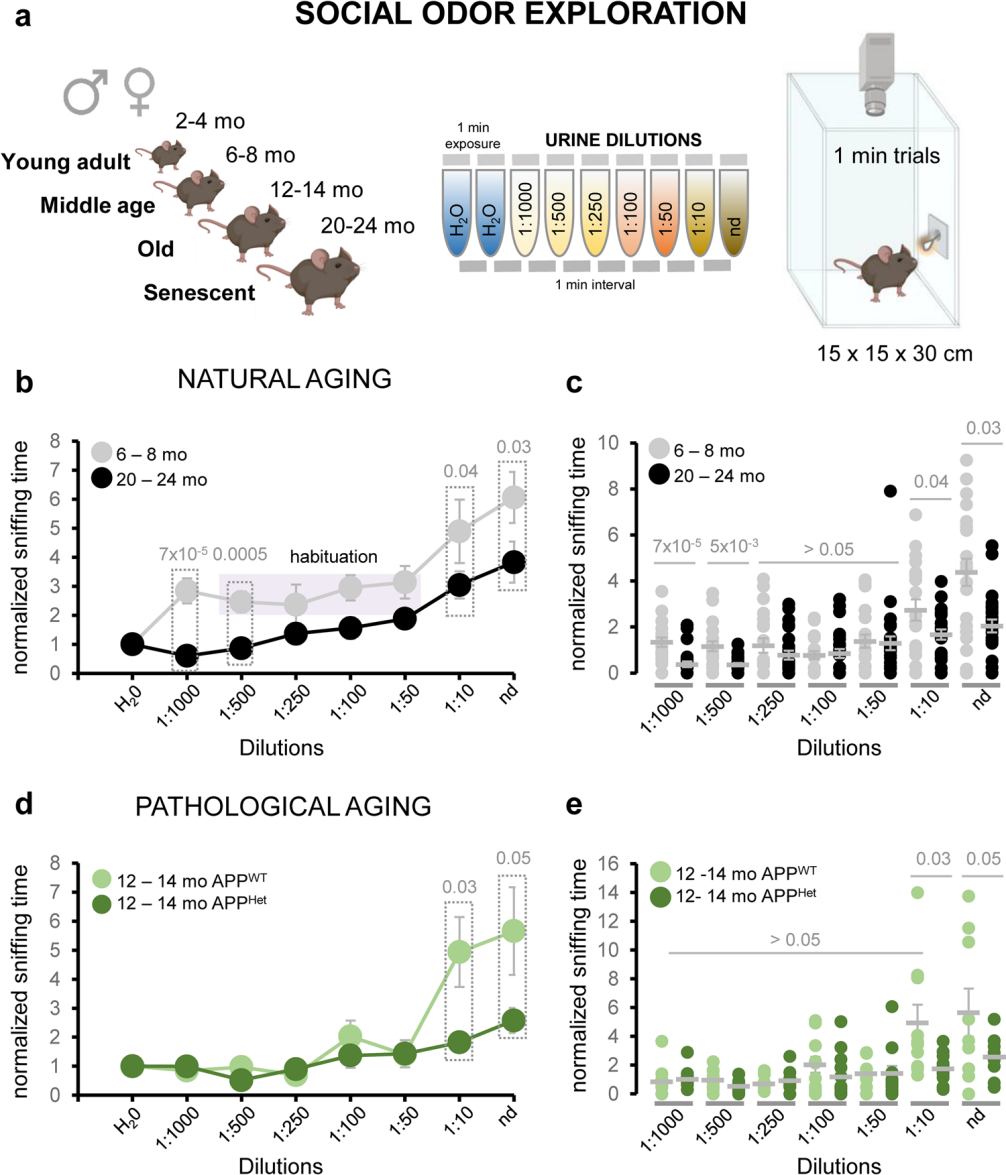
Natural and pathological aging reduces exploration to social odors. **a** Schematics of the olfactory test used in this study in which urine dilutions are presented as a social signal. **b** Average of the sniffing time of urine serial dilutions normalized to the exploration time of the vehicle (water) of adult and aged wild-type mice. A typical habituation indicated by a purple box was observed in adult mice at intermediate dilutions (1:500, 1:250, 1:100; 1:50). **c** Dispersion plot of the normalized sniffing time of each urine dilution (nd (non-diluted); 1:10; 1:50; 1:100; 1:250; 1:500; 1:1000) for adult and aged wild-type mice. **d** Average of the sniffing time of urine dilutions normalized to the exploration time of the vehicle (water) of middle-aged APP^WT^ controls and middle-aged APP/PS1^Het^ mice. **e** Dispersion plots of normalized sniffing time of middle-aged APP^WT^ controls and APP/PS1^Het^ mice. Grey lines in the dispersion plots indicate mean ± SEM. Data were analyzed by a one-way ANOVA with Tukey’s test to test multiple comparisons with more than one variable. $P \le 0.05$ was considered statistically significant. *P* values are indicated above the corresponding comparisons

To explore the impact of natural aging on social odor detection, we conducted an odor-evoked sniffing test in which serial dilutions of urine from young conspecifics of the opposite sex were presented to either male or female subjects over a range of different ages: young adult (2–4 mo.), middle age (6–8 mo.), old (12–14 mo.), and senescent mice (20–24 mo.) (Fig. 3a, see “Materials and Methods” for details on urine sample preparation). Our results indicated a significant reduction in the exploration time throughout various urine dilutions in comparison to adult wild-type mice (Fig. 3b, c; Supplementary Results—Table 7). Raw sniffing time data showed a similar decrease in the exploration of social odors in senescent animals across different dilutions (see details in Supplementary Figures 3 and 4).

Furthermore, senescent mice progressively increased the exploration time in parallel with odor concentration, whereas young adult, middle-aged, and old animals showed a habituation phase at intermediate dilutions (1:500, 1:250, 1:100, and 1:50), indicating effective odor detection and recognition capabilities [50]. Lastly, data analysis disaggregated by sex showed no sex differences in the reduction of urine exploration time (Supplementary Figure 5; but see [51] for an alternative view).

### Reduction in the Exploration of Social Information Is Accelerated in an Animal Model of Neurodegeneration

Next, we investigated whether pathological aging would alter the exploration of social olfactory cues despite no obvious effects on VSE structure or proliferative capacity. Social odor sensitivity tests in 1-year-old APP/PS1^Het^ mice revealed reduced sniffing times of low urine dilutions when compared to age-matched APP/PS1^WT^ control mice, suggesting that pathological aging accelerates the decline of exploration time of social odors (Fig. 3d, e; Supplementary Results—Table 8). Similar to senescent mice, these results were reproduced when raw sniffing time data were compared (Supplemental Figures 6 and 7).

### Natural Aging Mildly Reduces Neutral Odor Exploration

**Fig. 4:**
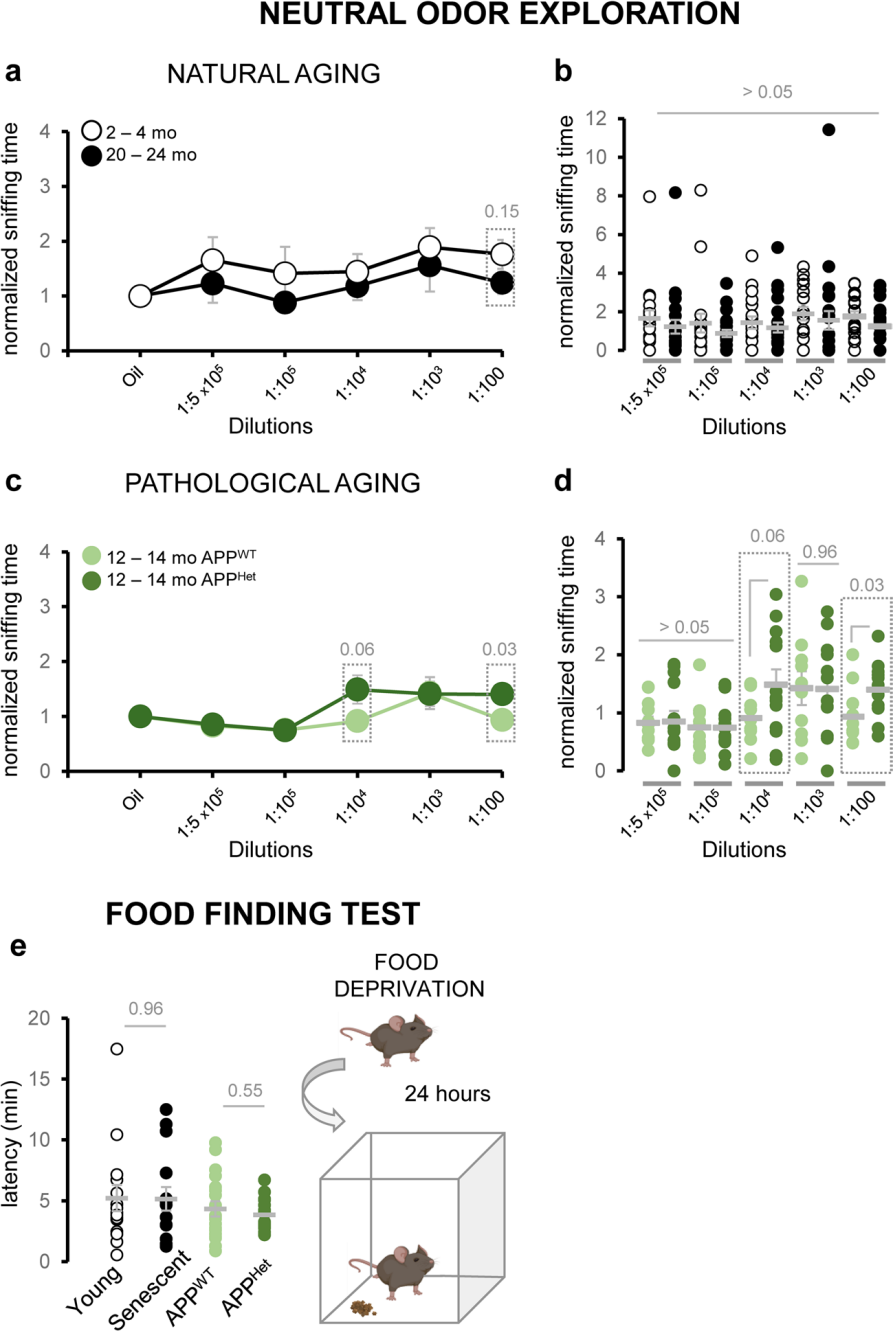
Aging and neurodegeneration impact social odor recognition more severely than other odor modalities. **a** Average of the sniffing time of young and aged mice in response to a neutral synthetic odor (IA) normalized to the vehicle (mineral oil). **b** Dispersion plots of the normalized sniffing time of young and aged mice in response to various IA dilutions. **c** Average of the sniffing time of middle-aged APP^WT^ and APP/PS1^Het^ mice in response to IA samples normalized to the vehicle (mineral oil). **d** Dispersion plots of normalized sniffing time of APP/PS1^WT^ and APP/PS1^Het^ mice in response to various IA dilutions. Note that APP/PS1^Het^ mice showed higher exploration times for the 1:10^4^ and 1:100 IA dilutions. **e** Food-deprived animals performed a food finding test (schematics on the right) which revealed equivalent latencies to find hidden food pellets as shown in the dispersion data plot (left panel). Thick grey lines in the dispersion plot indicate mean ± SEM. Data were analyzed by a one-way ANOVA with Tukey’s test to test multiple comparisons with more than one variable. $P \le 0.05$ was considered statistically significant. *P* values are indicated above the corresponding comparisons

Next, we asked whether non-social odor modalities were also affected in senescent and APP/PS1^Het^ animals. We investigated this question by analyzing the exploration time to both food and synthetic neutral odors. First, we exposed naturally aged and APP/PS1^Het^ mice to serial dilutions of IA, a synthetic banana-like odor of neutral valence when used at high dilutions [32, 33]. Consistent with previous studies, mice across all conditions showed significantly reduced responses to the neutral odorant than to urine (Fig. 4a–d) according to the higher social valence of urine over a synthetic odor [33, 52, 53]. Our results showed no significant differences on the exploration times of IA in senescent mice and a modest but significant increase, in the sniffing time of the 1:10^4^ and 1:100 IA dilutions in APP/PS1^Het^ animals (Fig. 4a–d; Supplementary Results—Tables 9 and 10; Supplementary Figure 8). Similarly, a FFT showed no significant differences in the latency to find food pellets after 24 h of food deprivation in either naturally aged or middle-aged APP/PS1^Het^ mice (Fig. 4e; Supplementary Results—Table 11). These results suggest that deficits in social exploration time at advanced stages of natural aging and in an animal model of AD are more pronounced than other odor modalities.

### Social Odor Discrimination and Habituation Are Reduced in Naturally Aged and APP/PS1Het Mice

**Fig. 5:**
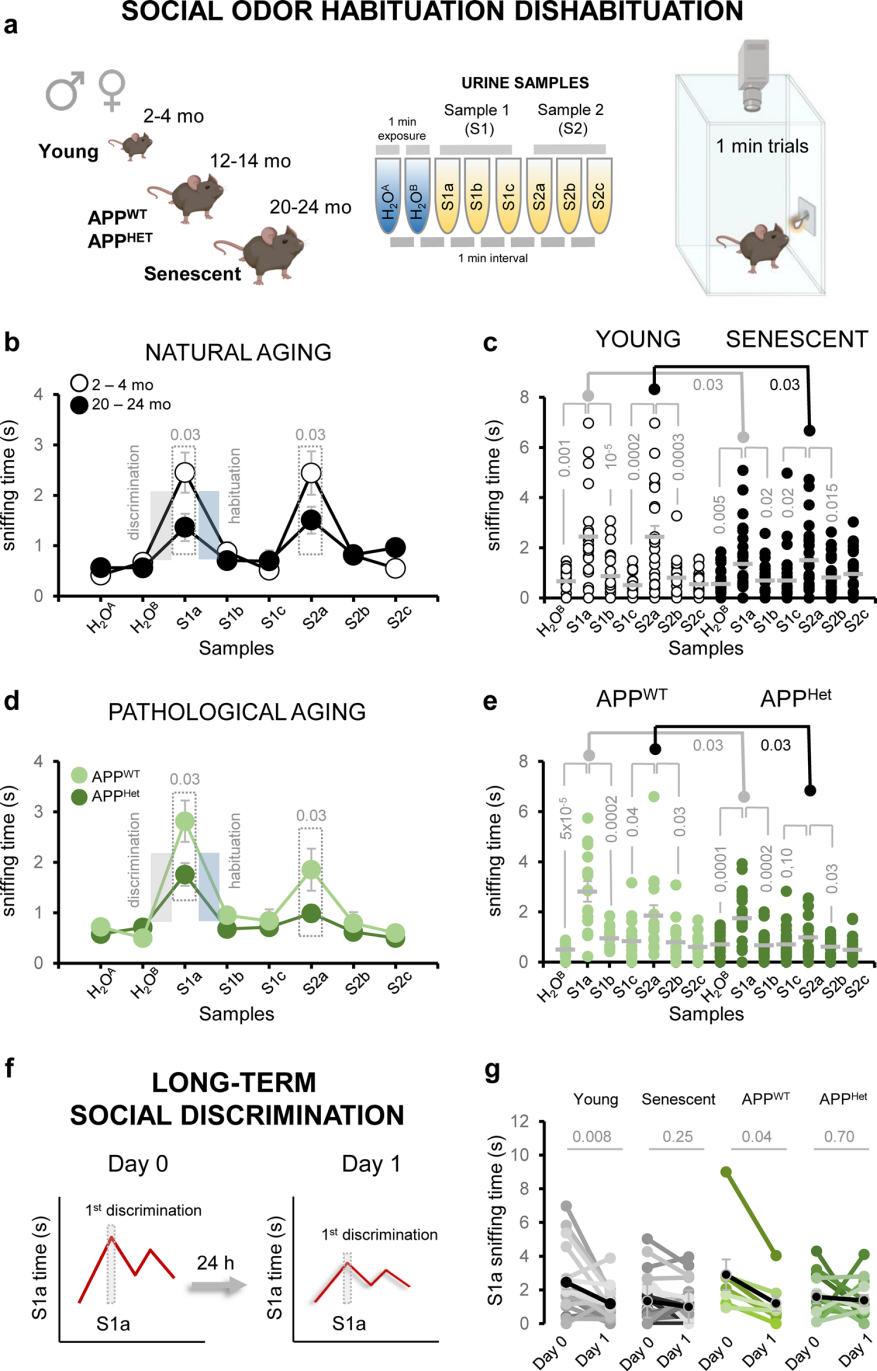
Social odor discrimination and habituation are reduced in naturally aged and APP/PS1^Het^ mice. **a** Schematics of the social habituation-dishabituation test used in this study: after habituation to the experimental cage, the animal was exposed to the same urine sample three times (S1a-c) which induces a typical increase in exploration time (1^st^ discrimination) to be followed by a reduction in the sniffing time (1^st^ habituation). Dishabituation induced by a urine sample from a new subject (S2a) elicits a second round of habituation–habituation (see “Materials and Methods” for details). **b** Average sniffing time of the social habituation-dishabituation test performed by young and aged wild-type mice. **c** Dispersion plot of the sniffing time of young and aged animals during the social habituation-dishabituation test. **d** Average sniffing time of the social habituation-dishabituation test performed by middle-aged APP/PS1^WT^ controls and age-matched APP/PS1^Het^ mice. **e** Dispersion plot of the sniffing time of middle-aged APP/PS1^WT^ controls and APP/PS1^Het^ mice during the social habituation-dishabituation test. **f** The habituation-dishabituation test was modified to assess potential deficits in long-term discrimination due to natural or pathological aging by presenting the same S1a sample 24 h after. **g** Paired data corresponding to the sniffing time during the first and second S1a presentation (24 h later) is plotted for each condition. Thick lines in dispersion plots and black dots in **g** indicate mean ± SEM. Data were analyzed by a one-way ANOVA with Tukey’s test to test multiple comparisons with more than one variable. $P \le 0.05$ was considered statistically significant. *P* values are indicated above the corresponding comparisons

**Fig. 6:**
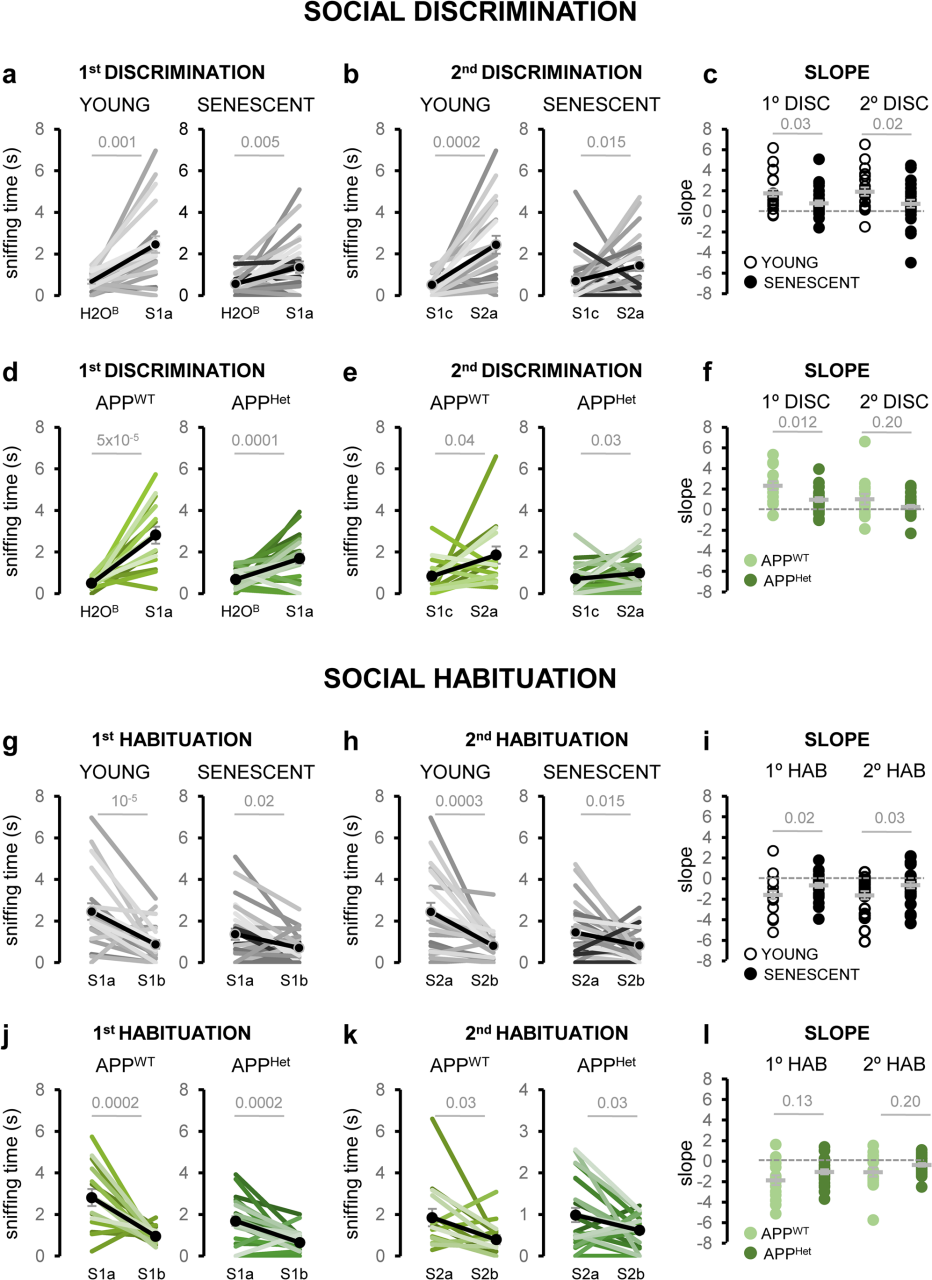
Analysis of the social discrimination and habituation decay during natural and pathological aging. **a** Paired data of the sniffing time of young and aged wild-type animals corresponding to the first discrimination phase (H_2_O-S1a). **b** Paired data of the sniffing time of young and aged wild-type animals corresponding to the second discrimination phase (S1c-S2a). **c** Dispersion plots of the slope values corresponding to the first and second discrimination phases of young and aged mice. **d** Paired data of the sniffing time of APP^WT^ and APP^Het^ mice corresponding to the first discrimination phase (H_2_O-S1a). **e** Paired data of the sniffing time of APP^WT^ and APP^Het^ mice corresponding to the second discrimination phase (S1c-S2a). **f** Dispersion plots of APP^WT^ and APP^Het^ mice corresponding to the slope values of the first and second discrimination phases. **g** Paired data of the sniffing time of young and aged wild-type animals corresponding to the first habituation phase (S1a-S1b). **h** Paired data of the sniffing time of the young and aged wild-type animals corresponding to the second habituation phase (S2a-S2b). **i** Dispersion plots of the slope values of young and aged mice corresponding to the first and second habituation phases. **j** Paired data of the sniffing time of APP^WT^ and APP^Het^ mice corresponding to the first habituation phase (S1a-S1b). **k** Paired data of the sniffing time of APP^WT^ and APP^Het^ mice corresponding to the second habituation phase (S2a-S2b). **l** Dispersion plots of the slope values of APP^WT^ and APP^Het^ mice corresponding to the first and second habituation phases. Black dots in **a**, **b**, **d**, **e**, **g**, **h**, **j**, **k** and thick lines in **c**, **f**, **i**, **l** indicate mean ± SEM. Data were analyzed by one-way ANOVA with Tukey’s test. $P \le 0.05$ was considered statistically significant. *P* values are indicated above the corresponding comparisons

To further investigate the impact of aging and neurodegeneration in the detection of social information, we performed a habituation–dishabituation test, which relies on the animal’s ability to discriminate novel smells [50]. For these experiments, young (2–4 mo.) and aged (20–24 mo.) wild-type animals were presented three consecutive replicates of urine samples from two different animals (S1a-c and S2a-c) (Fig. 5a). Aged animals were able to discriminate between urine sources, but showed reduced sniffing times during the first and second discrimination and habituation phases (Fig. 5b, c). Similarly, middle-aged APP/PS1^Het^ mice also exhibited reduced sniffing times in comparison to aged-matched APP/PS1^WT^ controls (Fig. 5d, e; Supplementary Results—Tables 12 and 13). Analysis of the slope values of the first and second discrimination and habituation phases revealed that senescent mice exhibited significant deficits during both rounds of social habituation–dishabituation, whereas APP/PS1^Het^ mouse impairments were apparent during the second phase of discrimination (Fig. 6; Supplementary Results—Tables 14 and 15).

Then, we adapted the habituation-dishabituation test to assay potential changes in long-term social odor memory (Fig. 5f). To this aim, animals were exposed to the same S1a urine sample 24 h after the first presentation. A reduction of the sniffing time during the first discrimination phase was interpreted as an indicator of memory. This reduction was clearly observed in young adults, but absent in naturally aged and 1-year-old APP/PS1^Het^ mice (Fig. 5g; Supplementary Results—Table 16), suggesting long-term social odor memory impairments during both natural and pathological aging.

### Age-Related Deficits in Social Discrimination and Habituation Are Not Influenced by Previous Experience

**Fig. 7:**
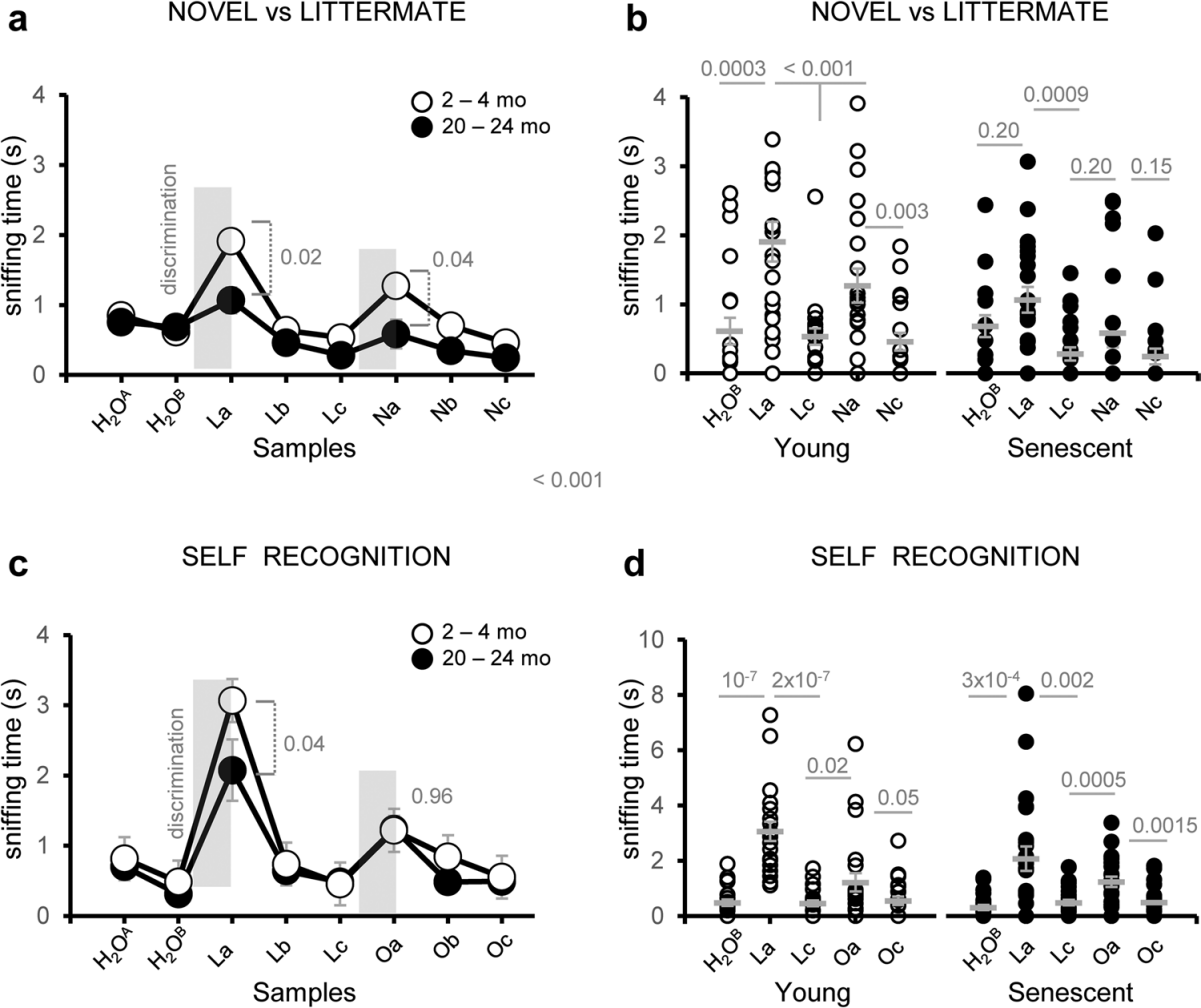
Age-related deficits in social discrimination and habituation are not influenced by previous experience. **a** Average sniffing time of social habituation-dishabituation test in response to odors from novel (N) or littermate (L) subjects of young and naturally aged mice. **b** Dispersion plot of the exploration time of young and senescent animals in response to novel or littermate urine samples. **c** Average sniffing time of social habituation-dishabituation test in response to littermate and animal’s own urine (O). **d** Dispersion plot of exploration time of young and naturally aged animals in response to their own or littermate urine samples. Thick grey lines in dispersion plots indicate mean ± SEM. Data were analyzed by a one-way ANOVA with Tukey’s test to test multiple comparisons with more than one variable. $P \le 0.05$ was considered statistically significant. *P* values are indicated above the corresponding comparisons

Next, we compared animals exposed to either familiar (littermate urine, L) or novel social odors (novel urine, N) in the habituation-dishabituation test (Fig. 7a). Aged animals performed poorly in the L-N dishabituation task (no significant increase in sniffing time), suggesting that the reduction in social odor discrimination and habituation is independent of previous experience.

Since impairments in social odor discrimination were more severe in naturally aged mice, we asked whether these deficits also extended to the recognition of animal’s own odors, as the loss of self-awareness is a disrupting symptom of common occurrence in senescent subjects [54, 55]. To this aim, animals were presented samples of their own urine (O) during the second discrimination phase of the habituation-dishabituation test (Fig. 7c, d). Our data indicated that animal’s own urine was effective to elicit a typical discrimination (dishabituation) response (Fig. 7; Supplementary Results—Tables 17 and 18), suggesting that self-recognition is preserved in senescent mice.

### Social Novelty Is Disrupted During Pathological Aging

**Fig. 8:**
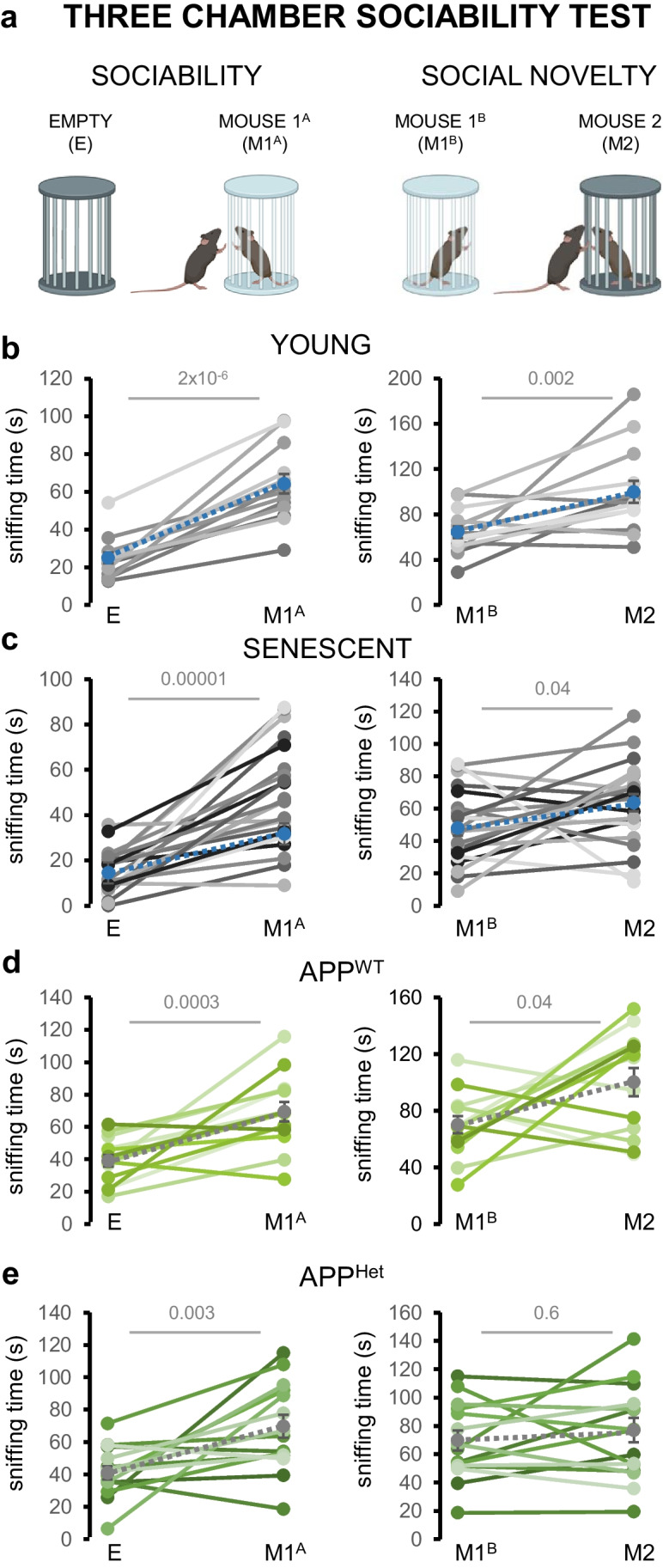
Social novelty is disrupted during pathological aging. **a** Schematics of the three-chamber test used in this study to test sociability and social novelty. In the sociability phase, the sniffing time of E and M1^A^ is compared. Social novelty is estimated by quantifying the sniffing time of exploring M1^B^ versus M2 (see “Materials and Methods”). **b** Paired data of the sniffing times of young wild-type mice during the sociability (E-M1^A^) and social novelty (M1^B^-M2) phases. **c** Paired data of the sniffing times of aged wild-type mice during sociability and social novelty. **d** Paired data of the sniffing times of middle-aged APP^WT^ mice during sociability and social novelty. **e** Paired data of the sniffing times of middle-aged APP^Het^ mice during sociability and social novelty. Colored dots in the paired plots indicate mean ± SEM. Data were analyzed by a one-way ANOVA with Tukey’s test to test multiple comparisons with more than one variable. $P \le 0.05$ was considered statistically significant. *P* values are indicated above the corresponding comparisons

Last, we explored whether the observed impairments in social odor exploration and discrimination may negatively impact social interaction in naturally aged and APP/PS1^Het^ mice. To this aim, we performed a three-chamber test [40, 56] (Fig. 8a) to assess general sociability and social novelty in senescent and APP/PS1^Het^ animals. Our data indicated a reduction in social novelty in middle-aged APP1/PS1^Het^ mice, which was not detected in 2-year-old animals (Fig. 8b–e; Supplementary Results—Tables 19 and 20). Although senescent mice did not show significant impairments in either sociability or social novelty, they exhibited a mild increase in the latency to approach M1 during the sociability phase (Supplementary Figure 9), consistent with the overall decrease in the exploration time of social odors (Supplementary Figures 3 and 4). These findings exposed exacerbated deficits in an animal model of AD, suggesting a distinct impact of natural and pathological aging on the display of social behavior.

## Discussion

The quality of social life has been proposed as a predictive factor for developing dementia or mental illness [6, 57]. However, the impairment of social functions with age is poorly understood with some authors suggesting that social deficits might be the consequence of generalized brain impairments [58] or a symptom which might be developed independently [59, 60]. Our results revealed that both natural and pathological aging affects several key aspects of social information processing including the exploration of social odors and social odor discrimination and habituation, with no obvious disruption of other odor modalities, suggesting specific alterations that affect how the aged brain integrates social information. To gain insight into the mechanisms underlying these deficits, we explored the age-related adaptions of the VSE, a central gateway for pheromone-encoded information in mammals [10–12] and part of the accessory olfactory system whose aging process has been largely overlooked in contrast to other aspects of olfaction [61].

Our data showed VSE alterations during natural aging but not in APP/PS1^Het^ mice, a standardized animal model of AD. Whereas the VSE of APP/PS1^Het^ mice maintained stable neurogenic capabilities, senescent wild-type animals showed a reduction in proliferative and stem cells (PCNA^+^ and Sox2^+^ cells) in the marginal VSE in comparison to young animals (Fig. 2), which in turn could explain the reduction of mature OMP^+^ neurons, SCL sustentacular Sox2^+^ cells, and organ volume (Fig. 1), although this may not imply a total elimination of proliferative capacities in old mice [18]. Our findings are consistent with two previous studies [18, 23], which reported an overall thinning of the vomeronasal sensory epithelium [23] and reduced VSE neurogenesis in the marginal zone of 2-year-old animals [18]. Here, we provide novel information from a commonly used AD animal model (APP/PS1^Het^), revealing an unexpected preservation of the VSE proliferative capabilities in middle-aged APP/PS1^Het^ mice. These results contrast with the reduction of SVZ neurogenesis reported in similar animal models [62–65], suggesting that VSE neurogenesis might be less susceptible to lesions (see [18]) and pathological conditions than other proliferative areas.

A relevant aspect of our work is that most previous studies addressing olfactory decline employed synthetic or neutral odors, thus remaining uncertain the specific effect of healthy and diseased aging in the recognition of social cues. To gain insight into this question, we investigated the exploration time and the habituation-dishabituation response to social odors (urine) (Figs. 3, 5, and 6). Our findings revealed that despite the distinctive effects of natural and pathological aging on VSE structure and cell composition, both processes impaired the exploration of socio-sexual cues, social discrimination-habituation, and social behavior, suggesting fundamental differences in the mechanisms by which healthy and diseased aging impact social information processing.

Further experiments are needed to establish causality, but our results suggest that the observed VSE alterations in senescent mice underlie deficits in urine exploration time (Fig. 3, Supplementary Figs. 3 and 4) which could impair the processing of social information. VSE volume data from middle-aged APP/PS1^WT^ control animals suggest that VSE structural changes might appear around 1-year-old (Fig. 1a), although their functional consequences might not become apparent until advanced stages of aging (2-year-old). This scenario suggests a parsimonious VNO decay that matches the aging of other olfactory areas like the OB. As such, several studies have shown that several symptoms of OB aging like reduced regeneration rate of olfactory sensory neurons (OSNs), decreased number of synaptic contacts [66], expression loss of odorant receptor genes [67, 68], or changes in the OSN dynamic range [69] are only clearly detectable in 2-year-old mice. This evidence suggests that although age-related changes may start earlier in the olfactory system [21], functional and behavioral deficits may exhibit a late onset, suggesting compensatory mechanisms to preserve the processing of olfactory cues relevant for the survival of aged animals [70, 71]. In contrast to the natural steady decline, pathological conditions may accelerate functional deficits even in the absence of peripheral organs modifications, suggesting alterations in the central processing of social information. As such, defects in the exploration of social odors were found exacerbated in middle-aged APP/PS1^Het^ mice (Fig. 3d, e), a condition likely to aggravate frailty and reduce life expectancy in these animals (as observed for APP/PSEN1^Het^ in our experimental conditions, see “Materials and Methods” for details).

Furthermore, results from the long-term social habituation-dishabituation test (S1a urine sample presentation after 24 h; Fig. 5f, g) revealed significant impairments in both 2-year-old wild-type animals and middle-aged APP/PS1^Het^ mice, suggesting that in parallel to sensory decline, the downstream pathways involved in social cue recognition may be affected during both natural and pathological aging. To control for a potential contribution of novelty in the social habituation-dishabituation test, we presented urine samples from littermate and novel animals finding similar deficits in discrimination (Fig. 7). Interestingly, although senescent animals showed severe discrimination deficits, they preserved the ability to differentiate between urine from a novel subject and their own, preventing to further explore the underlying mechanisms of subjective perception loss, a disrupting symptom of senescence and dementia which currently lacks appropriate animal models for preclinical studies [72].

Age-related decline of olfactory detection is likely to occur due to alterations of both the main olfactory epithelium and the VNO [67, 73], thus potentially affecting other odor modalities. Here, we sought to explore this question by performing odor-evoked sniffing tests employing social and neutral synthetic odors (Fig. 4). Quantification of the exploration time across various IA dilutions exposed no significant differences between senescent and young mice and even enhanced responses in APP/PS1^Het^ mice. This mild effect in the exploration of IA is probably partly due to the animals’ lower interest in exploring neutral odors as compared to conspecifics urine, as previously shown [33, 52, 53]. Nonetheless, results from a FFT revealed no changes in the latency time to find the hidden food pellets (Fig. 4e) indicating that defects in social odor detection related to natural and pathological aging might be more severe than other odor modalities like food odors, which drive vital behaviors such as foraging and feeding, reported to be mainly preserved at old age [74]. In rodents, the differentiation between learned and innate responses is believed to be maintained in advanced processing stages [52, 75]. However, this categorization may be an oversimplification since MOB mitral-tufted cells have been found to project to both the piriform cortex (associated with learned responses) and the posterolateral cortical amygdala (associated with innate responses). Additionally, recent research indicates that the patterns of activity in the piriform cortex and posterolateral cortical amygdala are essentially identical in response to odors of different types (e.g., conspecifics and predators) and valences (e.g., aversive, neutral, and appetitive) [76]. Therefore, the impairments in social odor exploration and discrimination may rise from maladaptations of the VNO-AOB axis, consistent with the observed decrease in AOB volume (Fig. 1b). Future studies should deepen into the anatomical and functional properties of the VNO-AOB circuit in order to obtain a complete picture on how healthy and pathological aging affects social information processing.

Finally, we sought to determine how the age-related defects in social odor sensitivity, discrimination, and memory shaped social behavior. Results from a three-chamber test (Fig. 8) indicated that sociability was overall preserved in senescent and APP/PS1^Het^ animals. In contrast, social novelty was found impaired in APP/PS1^Het^ mice consistently with previous studies [77–79]. However, it is important to note that our testing conditions in which the empty pencil cup is presented during the habituation may have exacerbated the curiosity towards M1^A^, potentially obscuring latent deficits in the social phase of the test.

Social novelty impairments in APP/PS1^Het^ mice could be linked to the decline in social discrimination observed in the habituation-dishabituation test (Figs. 5d, e and 6d, f) which may impair the recognition of M2 as a novel subject [80, 81]. Consistent with the overall decrease in the exploration time of social odors (Fig. 3, Supplementary Figs. 3 and 4), aged animals exhibited an increase in the latency to approach novel or familiar mice (M1^A^, M1^B^, M^2^) and a reduced number of approaches (Supplementary Fig. 9), which could be related to potential locomotion deficits that did not prevent and adequate performance of the three chamber test. These findings indicate that despite the reduction in social odor exploration and discrimination, overall sociability and novelty are majorly preserved in naturally aged mice. In contrast, the APP/PS1 neurodegenerative model shows a measurable impairment in social novelty, which might result from an overall problem in learning and memory as it is one of the main trademarks of AD and a common feature in transgenic mouse models for Aβ amyloidosis [25, 26, 78, 79]. This evidence supports the possibility that neuronal circuits underlying specific social functions (sociability vs. social memory) may be particularly susceptible to pathological aging.

## Conclusion

Olfactory deficits are a common symptom of natural and pathological aging. While multiple age-related changes of the olfactory sensory epithelium have been described, the aging of the pheromone detection system, a major gateway for social information, has been largely overlooked. This study reveals that whereas natural aging reduces VSE cell proliferation, mature sensory neurons and organ volume, a common animal model of AD exhibits normal proliferation capacities and no obvious morphological alterations. Despite exhibiting distinctive effects at the cellular level, both natural and pathological aging disrupt the detection of social odors in a more severe way than other odor modalities (i.e., neutral or food odors). Furthermore, social detection and social behavior impairments were exacerbated in APP/PS1^Het^ mice, indicating pathological aging impacts the downstream processing of social information even in the absence of VSE alterations.

## Supplementary Information

Below is the link to the electronic supplementary material.

Supplementary file1 (PDF 1300 KB)

Supplementary file2 (PDF 497 KB)

## Data Availability

The datasets supporting the conclusions of this article are included within the article and its additional files.

## Abbreviations

AD: Alzheimer’s disease
AOB: Anterior olfactory bulb
APP: Amyloid precursor protein
C: VNO central zone
CTF: Corrected total fluorescence
FFT: Food finding test
I: VNO intermediate zone
IA: Isoamyl acetate
M: VNO marginal zone
MOB: Main olfactory bulb
MOE: Main olfactory epithelium
NSE: Non-sensory epithelium
OCT: Optimal cutting temperature
OMP: Olfactory marker protein
PCNA: Proliferative cell nuclear antigen
PS1: Presenilin 1
SCL: Supporting cell layer
Sox2: SRY-box transcription factor 2
VL: Vomeronasal lumen
VNO: Vomeronasal organ
VSE: Vomeronasal sensory epithelium
