# Supplementary material for: Natural and Pathological Aging Distinctively Impacts the Pheromone Detection System and Social Behavior
Source: Mol Neurobiol. 2023 May 2;60(8):4641–58. doi: 10.1007/s12035-023-03362-3 (PMC10293359; doi:10.1007/s12035-023-03362-3)
Supplement: Supplementary file 1 — Supplementary file1 (PDF 1300 KB) [file 12035_2023_3362_MOESM1_ESM.pdf]

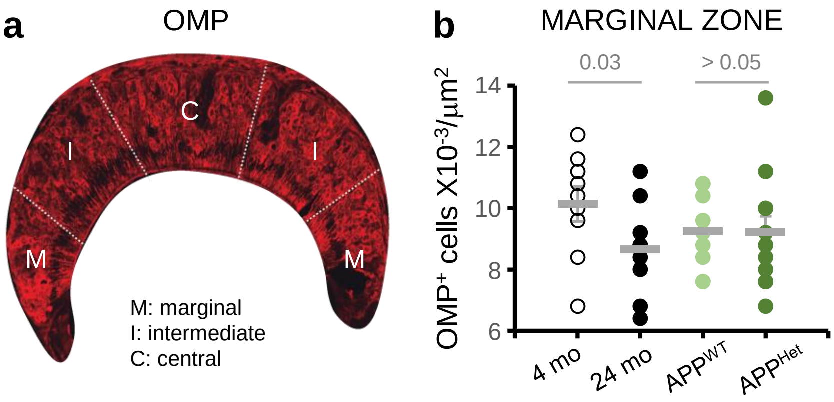

Supplementary Figure 1

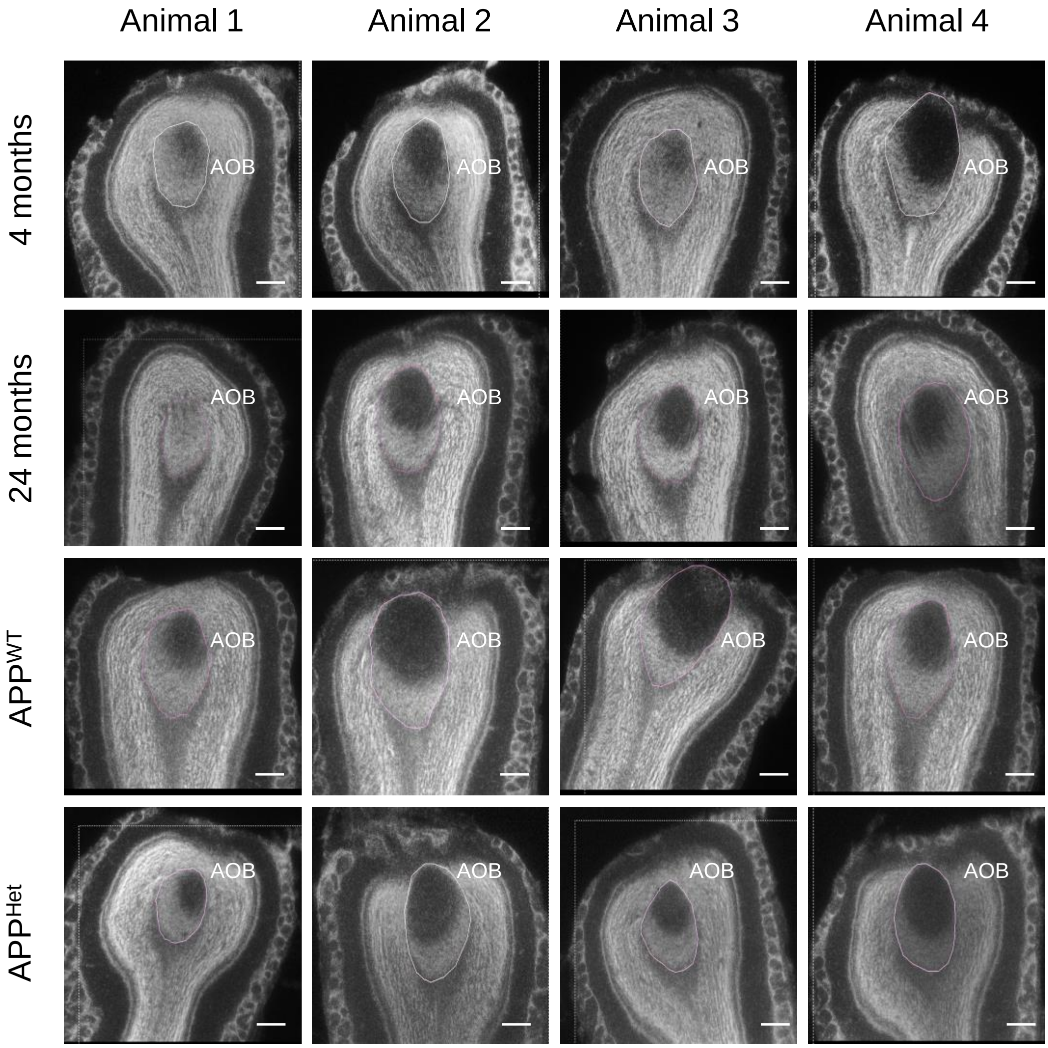

Supplementary Figure 2

SOCIAL ODOR EXPLORATION – NATURAL AGING

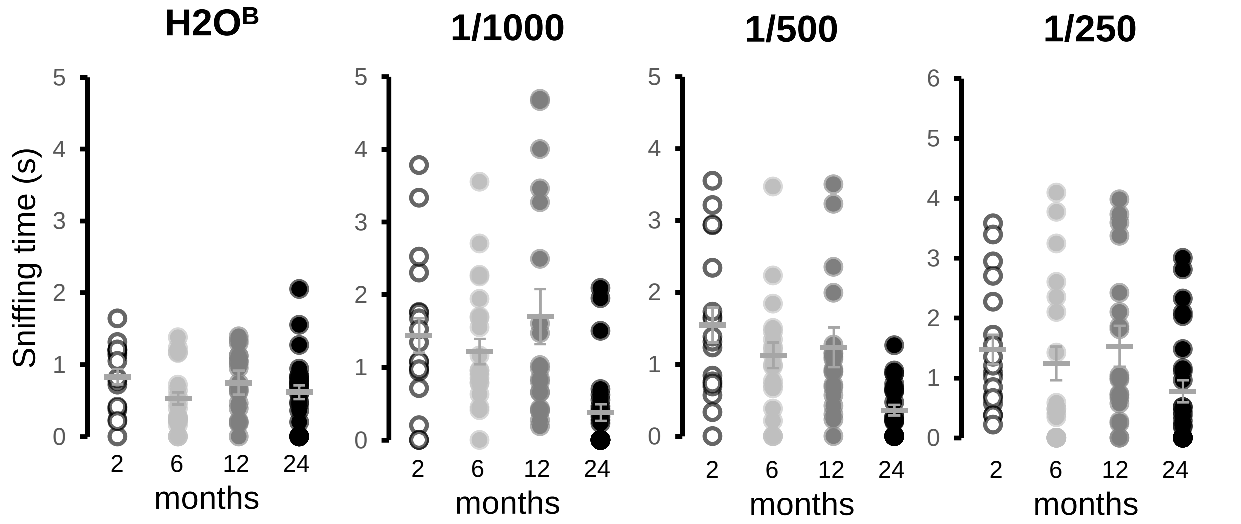

One-way ANOVA

H2O^B	
**Comparison**	***P***
4 m vs 8 m	0.02
4 m vs 12 m	ns
2 m vs 24 m	ns
8 m vs 12 m	ns
8 m vs 24 m	ns
12 m vs 24 m	ns

1:1000 urine dilution	
**Comparison**	***P***
4 m vs 8 m	ns
4 m vs 12 m	ns
4 m vs 24 m	0.0007
8 m vs 12 m	ns
8 m vs 24 m	0.0004
12 m vs 24 m	0.002

1:500 urine dilution	
**Comparison**	***P***
4 m vs 8 m	ns
4 m vs 12 m	ns
4 m vs 24 m	0.0002
44 m vs 12 m	ns
4 m vs 24 m	0.0005
12 m vs 24 m	0.0008

1:250 urine dilution	
**Comparison**	***P***
4 m vs 8 m	ns
4 m vs 12 m	ns
4 m vs 24 m	0.03
8 m vs 12 m	ns
8 m vs 24 m	ns
12 m vs 24 m	0.04

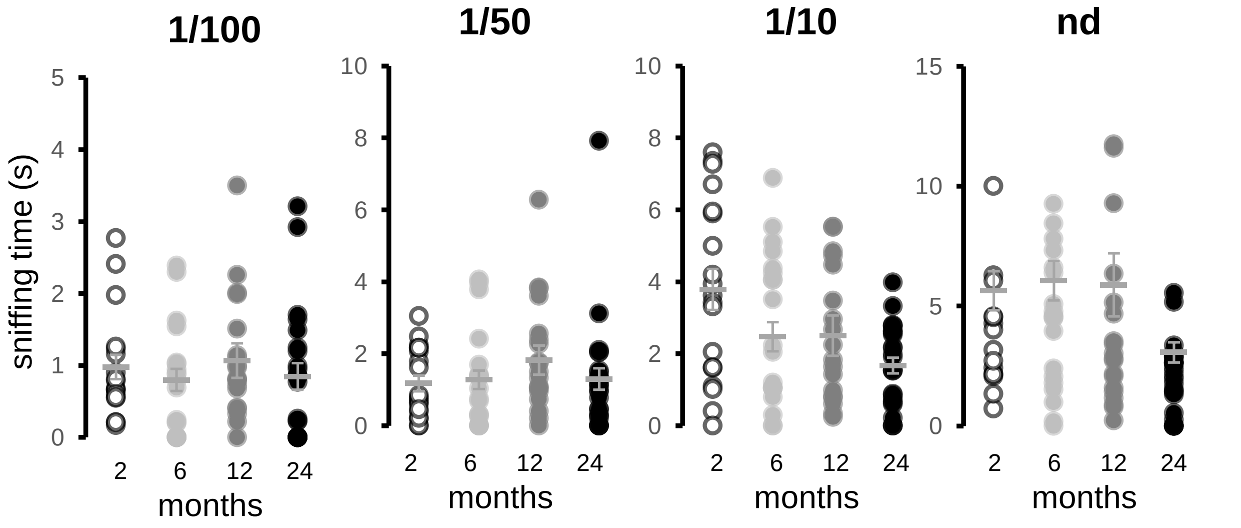

One-way ANOVA

1:100 urine dilution	
**Comparison**	***P***
4 m vs 8 m	ns
4 m vs 12 m	ns
4 m vs 24 m	ns
8 m vs 12 m	ns
8 m vs 24 m	ns
12 m vs 24 m	ns

1:50 urine dilution	
**Comparison**	***P***
4 m vs 8 m	ns
4 m vs 12 m	ns
4 m vs 24 m	ns
8 m vs 12 m	ns
8 m vs 24 m	ns
12 m vs 24 m	ns

1:10 urine dilution	
**Comparison**	***P***
4 m vs 8 m	0.08
4 m vs 12 m	0.04
4 m vs 24 m	0.002
8 m vs 12 m	ns
8 m vs 24 m	ns
12 m vs 24 m	ns

Non diluted	
**Comparison**	***P***
4 m vs 8 m	ns
4 m vs 12 m	ns
4 m vs 24 m	0.04
8 m vs 12 m	ns
8 m vs 24 m	0.003
12 m vs 24 m	0.02

dilution / age	*H_2O^B*	*1:000*	*1:500*	*1:250*	*1.100*	*1:50*	*1:10*	*nd*
	n	*n*	*n*	*n*	*n*	*n*	*n*	*n*
4 months	18	18	18	18	18	18	18	18
8 months	20	20	20	20	20	20	20	20
12 months	20	20	20	20	20	20	20	20
24 months	25	25	25	25	25	25	25	25

Supplementary Figure 3

SOCIAL ODOR EXPLORATION – NATURAL AGING

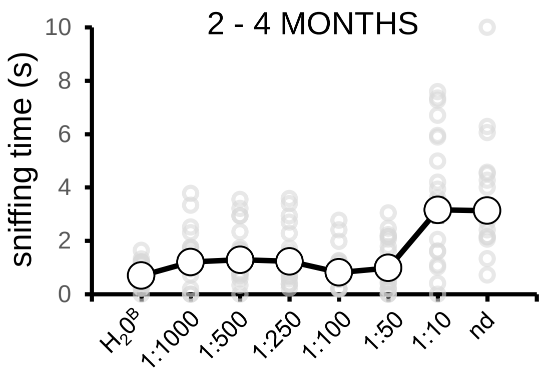

One Way ANOVA – Tukey test

2 months	
**Comparison**	***P***
H_2O^B vs 1/1000	0.91
H_2O^B vs 1/500	0.81
1/1000 vs 1/500	1.00
H_2O^B vs 1/250	0.88
1/250 vs 1/1000	1.00
1/250 vs 1/500	1.00
H_2O^B vs 1/100	0.99
1/100 vs 1/1000	0.97
1/100 vs 1/500	0.93
1/100 vs 1/250	0.99
H_2O^B vs 1/50	0.99
1/50 vs 1/1000	0.99
1/500 vs 1/50	0.99
1/250 vs 1/50	0.99

2 months	
**Comparison**	***P***
1/100 vs 1/50	0.99
H_2O^B vs 1/10	**1.5×10^{-7}**
1/10 vs 1/1000	**5.0×10^{-5}**
1/10 vs 1/500	**1.3×10^{-4}**
1/10 vs 1/250	**7.0×10^{-5}**
1/10 vs 1/100	**5.8×10^{-7}**
1/10 vs 1/50	**3.0×10^{-6}**
Nd vs H_2O^B	**1.0×10^{-6}**
Nd vs 1/1000	**2.2×10^{-4}**
Nd vs 1/500	**5.2×10^{-4}**
Nd vs 1/250	**3.0×10^{-4}**
Nd vs 1/100	**4.0×10^{-6}**
Nd vs 1/50	**1.8×10^{-5}**
Nd vs 1/10	1.00

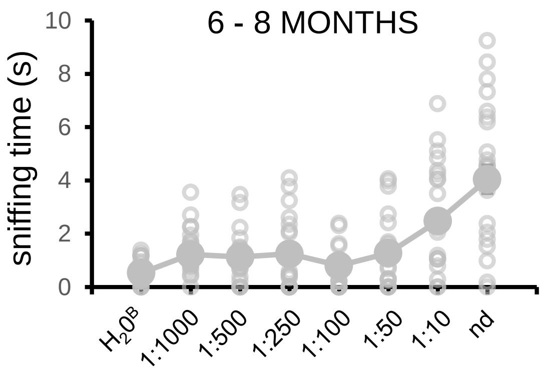

One Way ANOVA – Tukey test

6 months	
**Comparison**	***P***
H_2O^B vs 1/1000	0.63
H_2O^B vs 1/500	0.85
1/1000 vs 1/500	0.99
H_2O^B vs 1/250	0.82
1/250 vs 1/1000	0.99
1/250 vs 1/500	1.00
H_2O^B vs 1/100	0.99
1/100 vs 1/1000	0.94
1/100 vs 1/500	0.99
1/100 vs 1/250	0.98
H_2O^B vs 1/50	0.53
1/50 vs 1/1000	1.0
1/500 vs 1/50	0.99
1/250 vs 1/50	0.99

6 months	
**Comparison**	***P***
1/100 vs 1/50	0.90
H_2O^B vs 1/10	**6.4×10^{-5}**
1/10 vs 1/1000	0.07
1/10 vs 1/500	**0.02**
1/10 vs 1/250	**0.03**
1/10 vs 1/100	**0.0016**
1/10 vs 1/50	0.07
Nd vs H_2O^B	**2.2×10^{-8}**
Nd vs 1/1000	**8.3×10^{-8}**
Nd vs 1/500	**4.1×10^{-8}**
Nd vs 1/250	**$4,3 \times 10^{-8}$**
Nd vs 1/100	**4.5×10^{-8}**
Nd vs 1/50	**7.0×10^{-8}**
Nd vs 1/10	**0.01**

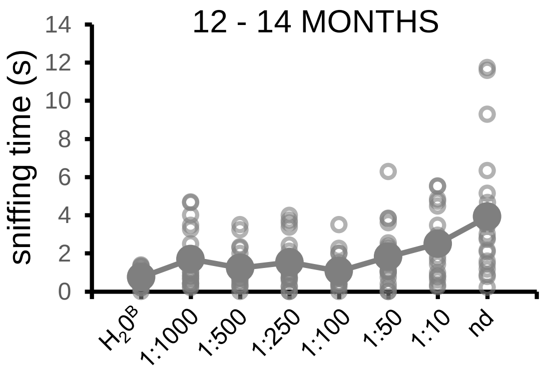

One Way ANOVA – Tukey test

12 months	
**Comparison**	***P***
H_2O^B vs 1/1000	0.67
H_2O^B vs 1/500	0.98
1/1000 vs 1/500	0.99
H_2O^B vs 1/250	0.85
1/250 vs 1/1000	0.99
1/250 vs 1/500	0.99
H_2O^B vs 1/100	0.99
1/100 vs 1/1000	0.94
1/100 vs 1/500	0.99
1/100 vs 1/250	0.99
H_2O^B vs 1/50	0.50
1/50 vs 1/1000	1.0
1/500 vs 1/50	0.95
1/250 vs 1/50	0.99

12 months	
**Comparison**	***P***
1/100 vs 1/50	0.85
H_2O^B vs 1/10	0.04
1/10 vs 1/1000	0.84
1/10 vs 1/500	0.31
1/10 vs 1/250	0.67
1/10 vs 1/100	0.17
1/10 vs 1/50	0.92
Nd vs H_2O^B	**1.3×10^{-6}**
Nd vs 1/1000	0.002
Nd vs 1/500	**7.2×10^{-5}**
Nd vs 1/250	**8.0×10^{-4}**
Nd vs 1/100	**2.0×10^{-5}**
Nd vs 1/50	0.005
Nd vs 1/10	0.20

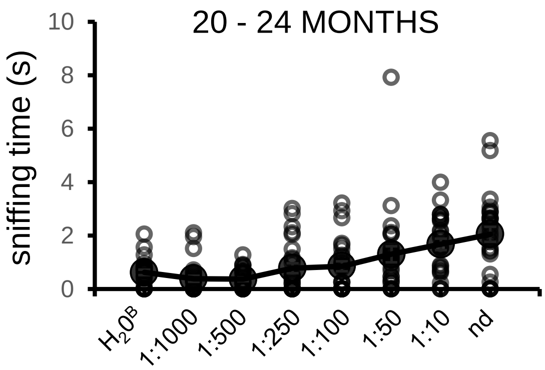

One Way ANOVA – Tukey test

24 months	
**Comparison**	***P***
H_2O^B vs 1/1000	0.99
H_2O^B vs 1/500	0.98
1/1000 vs 1/500	1.0
H_2O^B vs 1/250	0.99
1/250 vs 1/1000	0.87
1/250 vs 1/500	0.84
H_2O^B vs 1/100	0.99
1/100 vs 1/1000	0.73
1/100 vs 1/500	0.69
1/100 vs 1/250	1.0
H_2O^B vs 1/50	0.25
1/50 vs 1/1000	0.03
1/500 vs 1/50	0.03
1/250 vs 1/50	0.6

24 months	
**Comparison**	***P***
1/100 vs 1/50	0.76
H_2O^B vs 1/10	0.007
1/10 vs 1/1000	**3.4×10^{-4}**
1/10 vs 1/500	**2.6×10^{-4}**
1/10 vs 1/250	0.04
1/10 vs 1/100	0.08
1/10 vs 1/50	0.88
Nd vs H_2O^B	**3.4×10^{-5}**
Nd vs 1/1000	**8.4×10^{-7}**
Nd vs 1/500	**6.3×10^{-7}**
Nd vs 1/250	**$4,6 \times 10^{-4}$**
Nd vs 1/100	0.001
Nd vs 1/50	0.14
Nd vs 1/10	0.89

Supplementary Figure 4

SOCIAL ODOR EXPLORATION

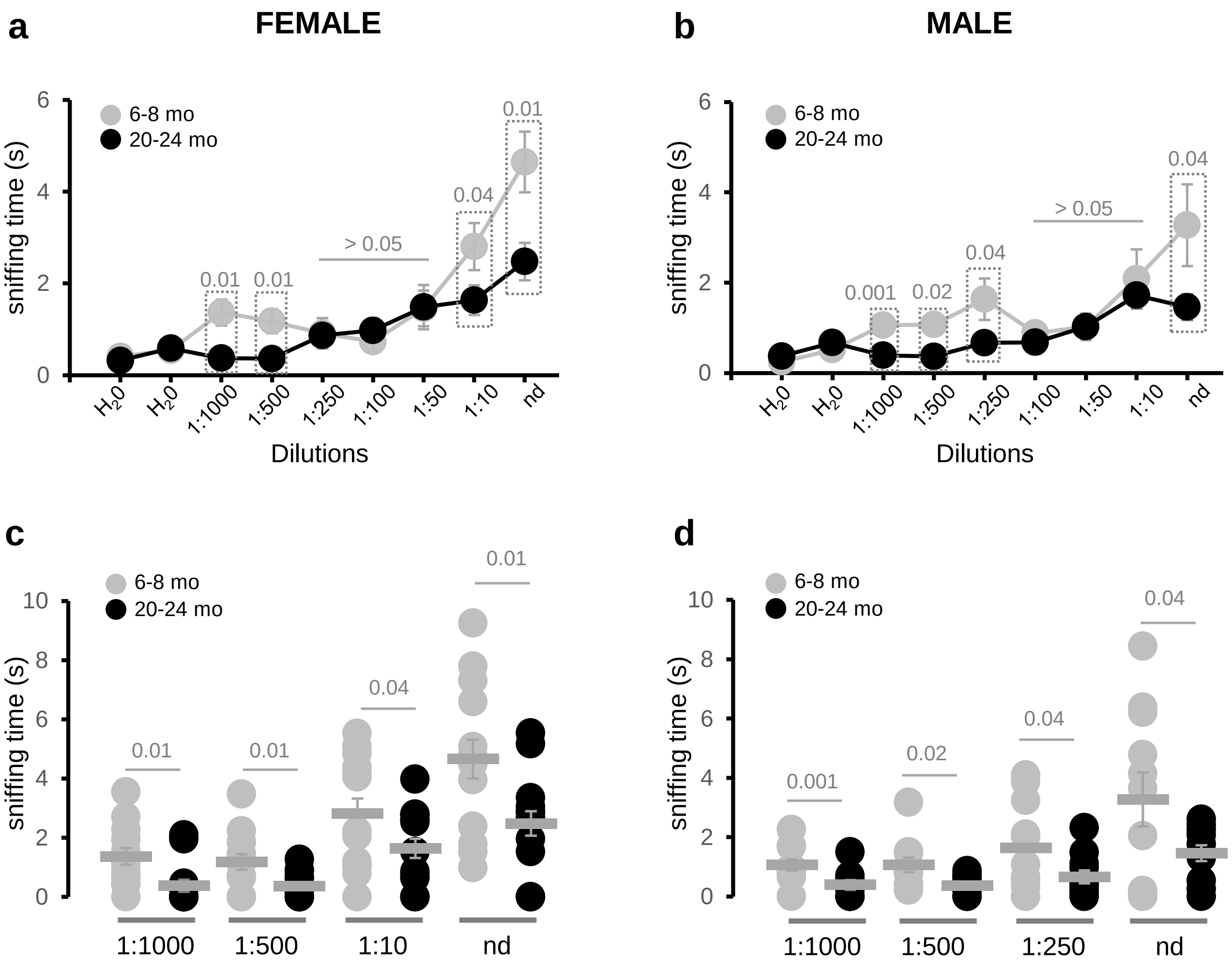

Supplementary Figure 5

SOCIAL ODOR EXPLORATION - PATHOLOGICAL AGING

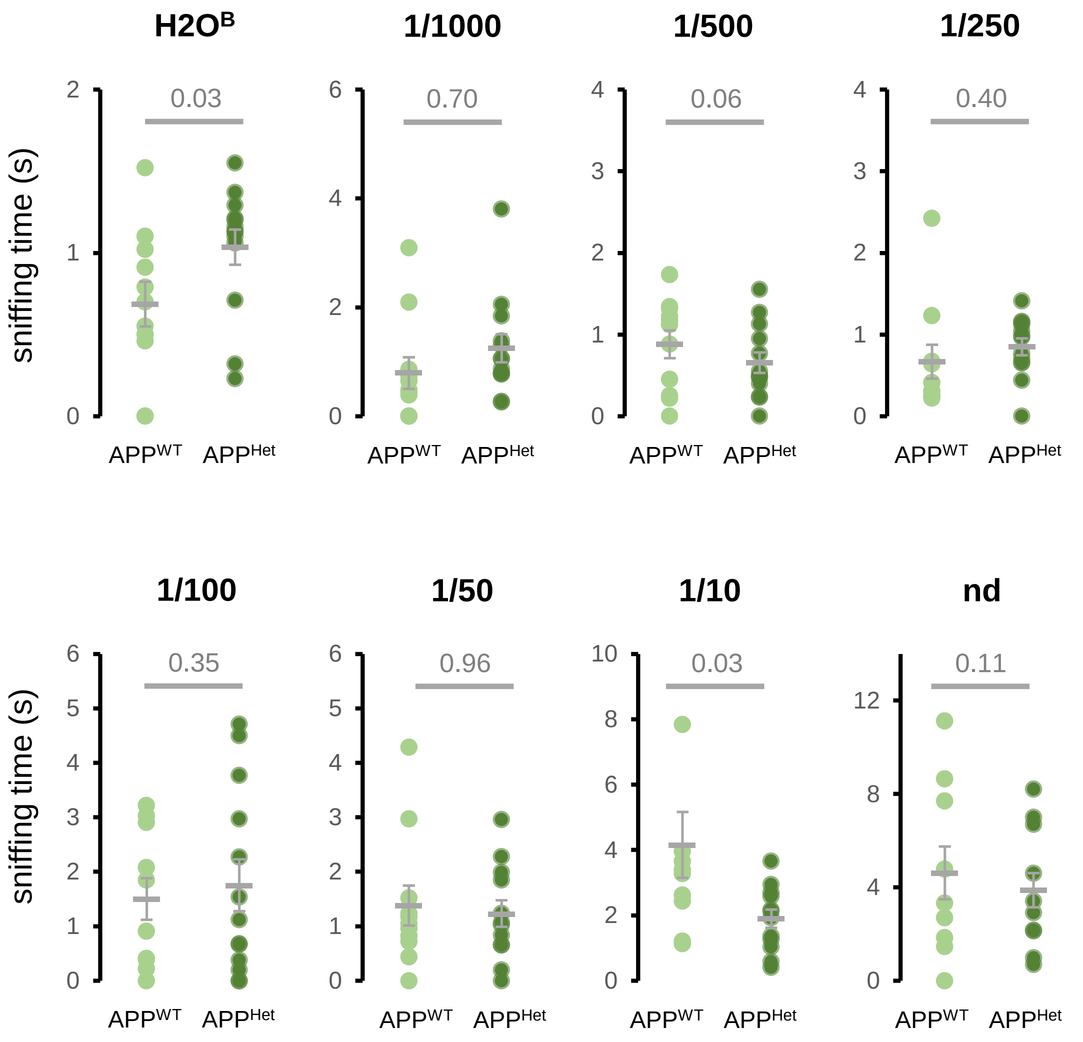

dilution / genotype	H_2O_B	1:1000	1:500	1:250	1.100	1:50	1:10	nd
	n	n	n	n	n	n	n	n
APP^{WT}	11	11	11	11	11	11	11	11
APP^{Het}	12	12	12	12	12	12	12	12

Supplementary Figure 6

SOCIAL ODOR EXPLORATION – PATHOLOGICAL AGING

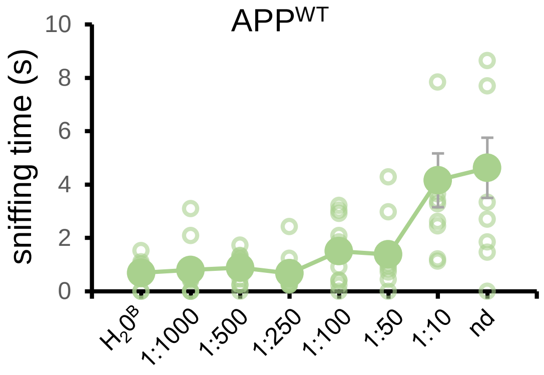

One Way ANOVA – Tukey test

APP^{WT}	
**Comparison**	***P***
H_2O^B vs 1/1000	1.00
H_2O^B vs 1/500	1.00
1/1000 vs 1/500	1.00
H_2O^B vs 1/250	1.00
1/250 vs 1/1000	1.00
1/250 vs 1/500	0.99
H_2O^B vs 1/100	0.97
1/100 vs 1/1000	0.99
1/100 vs 1/500	0.99
1/100 vs 1/250	0.97
H_2O^B vs 1/50	0.98
1/50 vs 1/1000	0.99
1/500 vs 1/50	0.99
1/250 vs 1/50	0.98

APP^{WT}	
**Comparison**	***P***
1/100 vs 1/50	1.00
H_2O^B vs 1/10	0.001
1/10 vs 1/1000	0.002
1/10 vs 1/500	0.003
1/10 vs 1/250	0.002
1/10 vs 1/100	0.04
1/10 vs 1/50	0.02
Nd vs H_2O^B	**3.1 x10^{-4}**
Nd vs 1/1000	**5.0 x10^{-4}**
Nd vs 1/500	**7.2 x10^{-4}**
Nd vs 1/250	**4.1 x10^{-4}**
Nd vs 1/100	0.01
Nd vs 1/50	0.005
Nd vs 1/10	0.99

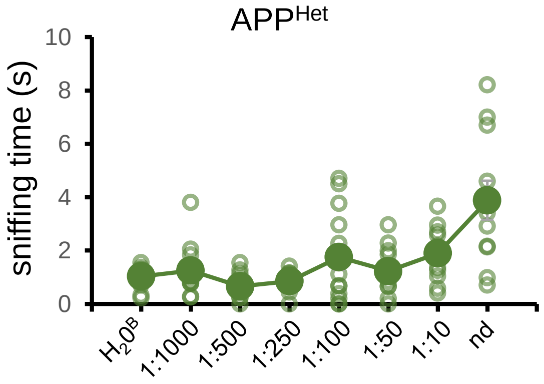

One Way ANOVA – Tukey test

APP^{Het}	
**Comparison**	***P***
H_2O^B vs 1/1000	0.99
H_2O^B vs 1/500	0.99
1/1000 vs 1/500	0.91
H_2O^B vs 1/250	0.99
1/250 vs 1/1000	0.99
1/250 vs 1/500	0.99
H_2O^B vs 1/100	0.79
1/100 vs 1/1000	0.96
1/100 vs 1/500	0.30
1/100 vs 1/250	0.99
H_2O^B vs 1/50	0.99
1/50 vs 1/1000	1.00
1/500 vs 1/50	0.93
1/250 vs 1/50	0.99

APP^{Het}	
**Comparison**	***P***
1/100 vs 1/50	0.95
H_2O^B vs 1/10	0.63
1/10 vs 1/1000	0.88
1/10 vs 1/500	0.18
1/10 vs 1/250	0.38
1/10 vs 1/100	0.99
1/10 vs 1/50	0.90
Nd vs H_2O^B	**6.6 x10^{-6}**
Nd vs 1/1000	**3.7 x10^{-5}**
Nd vs 1/500	**3.4 x10^{-7}**
Nd vs 1/250	**1.5 x10^{-6}**
Nd vs 1/100	0.002
Nd vs 1/50	**4.7 x10^{-5}**
Nd vs 1/10	0.005

Supplementary Figure 7

NEUTRAL ODOR EXPLORATION

NATURAL AGING

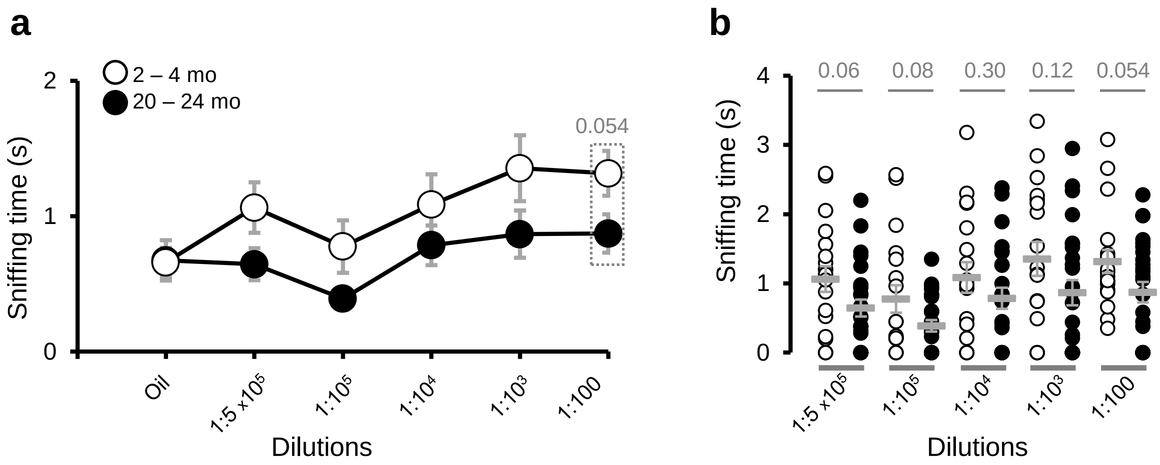

PATHOLOGICAL AGING

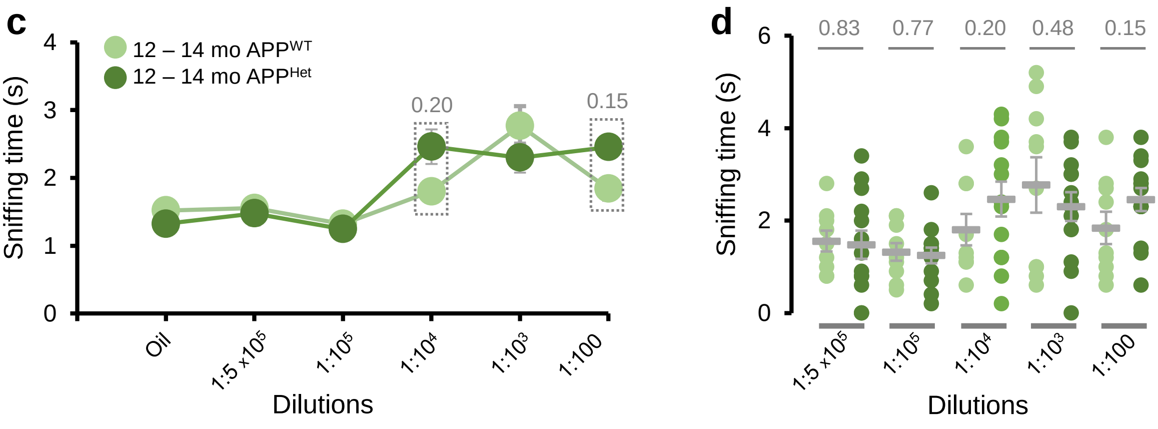

Supplementary Figure 8

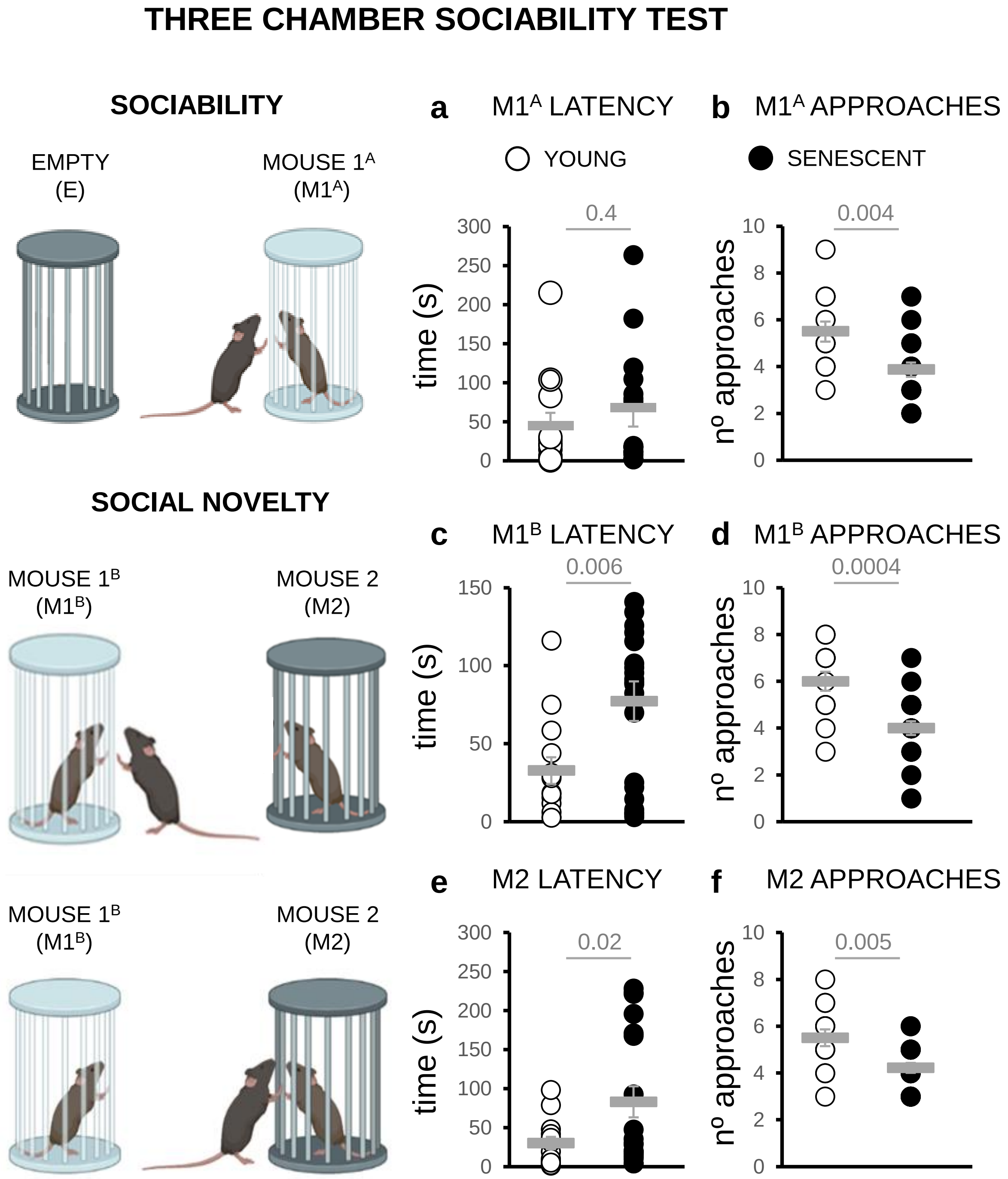

Supplementary Figure 9
