# Supplementary material for: Natural and Pathological Aging Distinctively Impacts the Pheromone Detection System and Social Behavior
Source: Mol Neurobiol. 2023 May 2;60(8):4641–58. doi: 10.1007/s12035-023-03362-3 (PMC10293359; doi:10.1007/s12035-023-03362-3)
Supplement: Supplementary file 2 — Supplementary file2 (PDF 497 KB) [file 12035_2023_3362_MOESM2_ESM.pdf]

Supplementary Results

Table 1. Figure 1a. VSE Volume (mm^3)

Age - Genotype	VSE Volume (mm^3)	N (mice)	One-way ANOVA Comparisons	One way ANOVA P value	Two-way ANOVA Age – Genotype interaction P value
4 months	0.190 ± 0.008	4	4 months vs 24 months	0.014	0.37
24 months	0.160 ± 0.007	4	APP^{WT} vs APP^{HET}	0.40	
1-year-old APP^{WT}	0.170 ± 0.007	4	4 months vs APP^{HET}	0.015	
1-year-old APP^{HET}	0.160 ± 0.008	4	24 months vs APP^{HET}	0.015	

Table 2. Figure 1e. Number of OMP+ cells / area

Age - Genotype	VSE region	OMP+ / um^2 x 10^{-3}	N (mice)	One way ANOVA Comparisons	One way ANOVA P value	Two-way ANOVA Age – Genotype Interaction P value
4 months	Anterior	10.69 ± 0.56	4	**Anterior**		**Anterior** 0.47
24 months	Anterior	7.80 ± 0.53	4	4 months vs 24 months	0.002	**Central** 0.87
1-year-old APP^{WT}	Anterior	9.50 ± 0.45	4	APP^{WT} vs APP^{HET}	0.21	**Posterior** 0.37
1-year-old APP^{HET}	Anterior	8.60 ± 0.53	4			
4 months	Medial	9.92 ± 0.23	4	**Central**		
24 months	Medial	7.70 ± 0.70	4	4 months vs 24 months	0.004	
1-year-old APP^{WT}	Medial	9.15 ± 0.78	4	APP^{WT} vs APP^{HET}	0.21	
1-year-old APP^{HET}	Medial	8.40 ± 0.40	4			
4 months	Posterior	9.15 ± 0.70	4	**Posterior**		
24 months	Posterior	7.90 ± 0.75	4	4 months vs 24 months	0.24	
1-year-old APP^{WT}	Posterior	9.53 ± 0.77	4	APP^{WT} vs APP^{HET}	0.96	
1-year-old APP^{HET}	Posterior	10.00 ± 0.84	4	24 mo vs APP^{HET}	0.16	

Table 3. Figure 1b AOB Volume (mm^3)

Age - Genotype	AOB Volume (mm^3)	N (mice)	One-way ANOVA Comparisons	One-way ANOVA P value	Two-way ANOVA Age – Genotype Interaction P value
4 months	0.48 ± 0.03	4	4 months vs 24 months	0.05	0.82
24 months	0.41 ± 0.03	4	APP^{WT} vs APP^{HET}	0.45	
1-year-old APP^{WT}	0.48 ± 0.04	4	4 months vs APP^{HET}	0.22	
1-year-old APP^{HET}	0.44 ± 0.03	4	24 months vs APP^{HET}	0.50	

Table 4. Figure 1f. Sox2 CTF / area - SCL

Age - Genotype	Sox2 CTF/um^2	N (mice)	One-way ANOVA Comparisons	One-way ANOVA P value	Two-way ANOVA Age – Genotype interaction P value
4 months	33.24 ± 2.43	4	4 months vs 24 months	$3x10^{-6}$	0.63
24 months	10.74 ± 1.32	4	APP^{WT} vs APP^{HET}	0.78	
1-year-old APP^{WT}	26.23 ± 3.95	4	24 months vs APP^{HET}	$2x10^{-8}$	
1-year-old APP^{HET}	27.37 ± 0.96	4			

Table 5. Figure 2b. Number of PCNA+ cells / area

Age - Genotype	VSE region	PCNA+ / um^2 x 10^{-3}	N (mice)	One-way ANOVA Comparisons	One-way ANOVA P value	Two-way ANOVA Age – Genotype Interaction P value
4 months	Anterior	2.26 ± 0.80	4	**Anterior**		**Anterior** 0.74
24 months	Anterior	0.75 ± 0.35	4	4 months vs 24 months	0.04	**Central** 0.014
1-year-old APP^{WT}	Anterior	0.73 ± 0.30	4	APP^{WT} vs APP^{HET}	0.01	**Posterior** 0.006
1-year-old APP^{HET}	Anterior	3.42 ± 0.82	4	24 months vs APP^{HET}	0.015	
4 months	Medial	2.15 ± 0.52	4	**Central**		
24 months	Medial	0.23 ± 0.13	4	4 months vs 24 mo	0.004	
1-year-old APP^{WT}	Medial	1.61 ± 0.32	4	APP^{WT} vs APP^{HET}	0.91	
1-year-old APP^{HET}	Medial	1.57 ± 0.20	4	24 months vs APP^{HET}	$4x10^{-6}$	
4 months	Posterior	3.04 ± 0.90	4	**Posterior**		
24 months	Posterior	0.17 ± 0.08	4	4 months vs 24 months	0.02	
1-year-old APP^{WT}	Posterior	2.42 ± 0.60	4	APP^{WT} vs APP^{HET}	0.56	
1-year-old APP^{HET}	Posterior	1.93 ± 0.57	4	24 months vs APP^{HET}	0.02	

Table 6. Figure 2c. $Sox2^+$ cells / area - VSE

Age - Genotype	$Sox2^+$ cells /um^2	N (mice)	One-way ANOVA Comparisons	One-way ANOVA P value	Two-way ANOVA Age – Genotype Interaction P value
4 months	3.08 ± 0.40	4	4 months vs 24 months	0.05	0.53
24 months	2.37 ± 0.36	4	APP^{WT} vs APP^{HET}	0.07	
1-year old APP^{WT}	2.92 ± 0.38	4	24 months vs APP^{HET}	0.62	
1-year old APP^{HET}	2.15 ± 0.24	4			

Table 7. Figure 3 b-c. Social odor exploration. Natural aging

	**Normalized sniffing time (s)**		**One-way ANOVA P value**
**Dilution**	**Middle age N = 20 mice**	**Senescent N = 25 mice**	Middle age vs Senescent
1:1000	2.80 ± 0.43	0.60 ± 0.20	$7x10^{-5}$
1:500	2.50 ± 0.35	0.86 ± 0.20	0.0005

1:250	2.36 ± 0.70	1.37 ± 0.36	0.20
1:100	2.95 ± 0.43	1.56 ± 0.37	0.05
1:50	3.14 ± 0.55	1.90 ± 0.33	0.10
1:10	4.90 ± 1.10	3.04 ± 0.50	0.04
ND	6.06 ± 0.88	3.83 ± 0.71	0.03

Table 8. Figure 3 d-e. Social odor exploration. Pathological aging

	**Normalized sniffing time (s)**		**P value**
**Dilution**	**APP^{WT} N = 11 mice**	**APP^{HET} N = 12 mice**	APP^{WT} vs APP^{HET}
1:1000	0.84 ± 0.31	1.00 ± 0.20	0.70
1:500	0.97 ± 0.20	0.52 ± 0.10	0.06
1:250	0.70 ± 0.14	0.90 ± 0.17	0.40
1:100	2.03 ± 0.55	1.37 ± 0.43	0.35
1:50	1.40 ± 0.26	1.43 ± 0.46	0.96
1:10	4.94 ± 1.21	1.82 ± 0.27	0.03
ND	5.65 ± 1.51	2.58 ± 0.43	0.05

**Dilution**	**Two-way ANOVA Age – Genotype Interaction P value**
1:1000	0.02
1:500	0.0003
1:250	0.015
1:100	0.88
1:50	0.53
1:10	0.51
ND	0.33

Table 9. Figure 4 a-b. Neutral odor exploration. Natural aging

	**Normalized sniffing time (s)**		**One-way ANOVA P value**
**Dilution**	**Young N = 18 mice**	**Senescent N = 20 mice**	Young vs Senescent
$1{:}5x10^5$	1.65 ± 0.42	1.23 ±0.35	0.50
$1{:}10^5$	1.41 ± 0.50	0.90 ± 0.20	0.33
$1{:}10^4$	1.44 ± 0.32	1.20 ±0.26	0.54
$1{:}10^3$	1.90 ± 0.35	1.56 ± 0.50	0.60
1:100	1.76 ± 0.26	1.24 ± 0.20	0.15

Table 10. Figure 4 c-d. Neutral odor exploration. Pathological aging

	**Normalized sniffing time (s)**		**One-way ANOVA P value**
**Dilution**	**APP^{WT} N = 10 mice**	**APP^{HET} N = 13 mice**	APP^{WT} vs APP^{HET}
$1{:}5x10^5$	0.82 ± 0.11	0.85 ± 0.18	0.90
$1{:}10^5$	0.75 ± 0.15	0.75 ± 0.11	0.97
$1{:}10^4$	0.91 ± 0.13	1.50 ± 0.25	0.06
$1{:}10^3$	1.42 ± 0.30	1.40 ± 0.22	0.96
1:100	0.93 ±0.16	1.40 ± 0.12	0.03

Dilution	Two-way ANOVA<br>Age – Genotype<br>Interaction<br>P value
1:5x10^5	0.50
1:10^5	0.71
1:10^4	0.99
1:10^3	0.80
1:100	0.46

Table 11. Figure 4e. Food finding test latency (min)

Condition	Latency (min)	N (mice)	One-way ANOVA Comparisons	One-way ANOVA P value	Two-way ANOVA Age-Genotype interaction P value
4 months	5.22 ± 1.08	15	4 mo vs 24 mo	0.96	0.13
24 months	5.15 ± 0.95	15	APP^{WT} vs APP^{HET}	0.55	
1-year-old APP^{WT}	3.87 ± 0.32	16			
1-year-old APP^{HET}	4.27 ± 0.58	20			

Table 12. Figure 5 b, c. Figure 6 a, b, g, h. Social habituation – Natural aging

	Sniffing time (s)		One-way ANOVA P value
**Test phase**	**Young N = 21 mice**	**Senescent N = 26 mice**	Young vs Senescent
Water control	0.67 ± 0.10	0.56 ± 0.11	0.30
S1a	2.45 ± 0.40	1.36 ± 0.27	0.03
S1b	0.87 ± 0.20	0.70 ± 0.14	0.50
S1c	0.51 ± 0.10	0.70 ± 0.21	0.44
S2a	2.44 ± 0.43	1.51 ± 0.27	0.03
S2b	0.81 ± 0.15	0.82 ± 0.14	0.94
S2c	0.55 ± 0.11	0.96 ± 0.18	0.06

Table 13. Figure 5 d, e. Figure 6 d, e, j, k. Social habituation – Pathological aging

	Sniffing time (s)		One way-ANOVA P value
**Test phase**	**APPWT N = 15 mice**	**APPHET N = 24 mice**	APP^{WT} vs APP^{HET}
Water control	0.50 ± 0.07	0.71 ± 0.09	0.70
S1a	2.81 ± 0.40	1.75 ± 0.22	0.03
S1b	0.95 ± 0.10	0.68 ± 0.10	0.08
S1c	0.84 ± 0.22	0.71 ± 0.13	0.62
S2a	1.85 ± 0.42	1.00 ± 0.17	0.03
S2b	0.80 ± 0.22	0.62 ± 0.08	0.50
S2c	0.60 ± 0.13	0.50 ± 0.09	0.53

Dilution	Two-way ANOVA<br>Age – Genotype<br>Interaction<br>P value
Water control	0.51
S1a	0.70
S1b	0.15
S1c	0.84
S2a	0.33
S2b	0.17

S2c	0.70

Table 14. Figure 6 c, i. Social habituation – discrimination slopes – Natural aging

	Slope		P value
**Test phase**	**Young N = 21 mice**	**Senescent N = 26 mice**	Middle age vs Senescent
1st discrimination	1.78 ± 0.36	0.80 ± 0.30	0.03
2nd discrimination	1.92 ± 0.40	0.75 ± 0.40	0.02
1st habituation	-1,57 ± 0.40	-0.65 ± 0.24	0.02
2nd habituation	-1.62 ± 0.40	-0.62 ± 0.32	0.03

Table 15. Figure 6 f, l. Social habituation – discrimination slopes – Pathological aging

	Slope		P value
**Test phase**	**APP^{WT} N = 15 mice**	**APP^{HET} N = 24 mice**	APP^{WT} vs APP^{HET}
1st discrimination	2.32 ± 0.43	0.97 ± 0.24	0.012
2nd discrimination	1.01 ± 0.51	0.27 ± 0.20	0.20
1st habituation	-1.86 ± 0.50	-1.03 ± 0.24	0.13
2nd habituation	-1.06 ± 0.44	-0.37 ± 0.18	0.20

**Test phase**	**Two-way ANOVA Age – Genotype Interaction P value**
1st discrimination	0.41
2nd discrimination	0.19
1st habituation	0.65
2nd habituation	0.007

Table 16. Figure 5 g. Long-term social discrimination

	Sniffing time (s)		One-way ANOVA P value	Two-way ANOVA Age – Genotype interaction P value
**Test phase**	**Day 0**	**Day 1**	**Day 0 vs Day 1**	0.98
4 months (n = 19 mice)	2.45 ± 0.07	1.20 ± 0.20	0.008	
24 months (n = 20 mice)	1.36 ± 0.30	1.12 ± 0.30	0.25	
1-year-old APP^{WT} (n = 8 mice)	3.12 ± 0.90	1.65 ± 0.60	0.04	
1-year-old APP^{HET} (n = 13 mice)	1.60 ± 0.40	1.40 ± 0.33	0.70	

Table 17. Figure 7. Social habituation – Novel vs littermate

	Sniffing time (s)		One-way ANOVA P value
**Test phase**	**Young N = 21 mice**	**Senescent N = 23 mice**	Young vs Senescent
Water control	0.61 ± 0.20	0.68 ± 0.16	0.70
La	1.91 ± 0.30	1.10 ± 0.20	0.02
Lc	0.53 ± 0.11	0.30 ± 0.09	0.3
Na	1.27 ± 0.25	0.60 ± 0.20	0.04
Nc	0.45 ± 0.13	0.24 ± 0.11	0.2

Table 18. Figure 7. Social habituation – Self recognition

	**Sniffing time (s)**		**One-way ANOVA P value**
**Test phase**	**Young N = 21 mice**	**Senescent N = 23 mice**	Young vs Senescent
Water control	0.48 ± 0.11	0.31 ± 0.09	0.70
La	3.06 ± 0.35	2.07 ± 0.43	0.04
Lc	0.45 ± 0.10	0.48 ± 0.10	0.64
Oa	1.22 ± 0.33	1.23 ± 0.18	0.96
Oc	0.84 ± 0.13	0.48 ± 0.13	0.90

Table 19. Figure 8 b, c. Three chamber sociability test – Natural aging

**Test phase**	**Sniffing time (s)**		**One-way ANOVA P value**
	**Young N = 14 mice**	**Senescent N = 22 mice**	
**Sociability**			**Empty vs M1^A**
Empty	25.06 ± 2.96	14.50 ± 1.90	Middle age: 2x10^{-6}
M1^A	64.35 ± 5.23	47.24 ± 4.60	Senescent: 1x10^{-5}
**Social novelty**			**M1^B vs M2**
M1^B	62.37 ± 5.01	49.15 ± 4.20	Middle age: 0.002
M2	99.93 ± 9.83	63.70 ± 5.30	Senescent: 0.04

Table 20. Figure 8 c,d. Three chamber sociability test – Pathological aging

**Test phase**	**Sniffing time (s)**		**One-way ANOVA P value**
	**APP^{WT} N = 14 mice**	**APP^{HET} N = 15 mice**	
**Sociability**			**Empty vs M1^A**
Empty	39.15 ± 3.63	41.00 ± 4.07	APP^{WT}: 0.0003
M1^A	69.40 ± 6.05	69.73 7.10	APP^{HET}: 0.003
**Social novelty**			**M1^B vs M2**
M1^B	71.30 ± 5.75	65.12 ± 6.70	APP^{WT}: 0.04
M2	93.07 ± 11.70	77.02 ± 8.43	APP^{HET}: 0.60

**Test phase**	**Two-way ANOVA Age – Genotype interaction P value**
**Sociability M1A - Empty**	0.96
**Social novelty M2 – M1B**	0.83
